# Algorithm-assisted individualized therapy design improves survival in a mouse model of triple-negative breast cancer

**DOI:** 10.1038/s41698-025-01245-5

**Published:** 2026-01-19

**Authors:** Balázs Gombos, Violetta Léner, Dániel András Drexler, Bence Czakó, Tamás Ferenci, Levente Kovács, Dániel Kiss, Pál Szabó, József Tóvári, Gergely Szakács, András Füredi

**Affiliations:** 1 Drug Resistance Research Group, Institute of Molecular Life Sciences, HUN - REN Research Center for Natural Sciences, Budapest, Hungary; 2 Semmelweis University, Doctoral School, Budapest, Hungary; 3 Department of Experimental Pharmacology and the National Tumor Biology Laboratory, National Institute of Oncology, Budapest, Hungary; 4 Physiological Controls Research Center, University Research and Innovation Center, Obuda University, Budapest, Hungary; 5 John von Neumann Faculty of Informatics, Obuda University, Budapest, Hungary; 6 Centre for Structural Study, HUN-REN Research Centre for Natural Sciences, Budapest, Hungary; 7 Center for Cancer Research, Medical University of Vienna, Vienna, Austria; 8 Microsystems Laboratory, Institute of Technical Physics and Materials Science, HUN-REN Centre for Energy Research, Budapest, Hungary

**Keywords:** Cancer, Computational biology and bioinformatics, Drug discovery, Oncology

## Abstract

Chemotherapy remains indispensable in the treatment of malignant tumors but is often limited by the prevailing “one size fits all” approach, which neglects inter-patient variablity in pharmacokinetics and treatment response, often resulting in suboptimal outcomes. In this study, we explored individualized chemotherapy protocols in a clinically relevant mouse model of breast cancer using a novel algorithm-assisted therapy design (AATD). Two strategies were applied: a two-stage computational therapy protocol designed to stabilize blood concentrations of pegylated liposomal doxorubicin (PLD); and a model-predictive approach that optimizes dosing based on individual tumor characteristics. Compared to the standard maximum tolerated dose protocol, AATD-based personalized chemotherapy, guided by real-time monitoring of treatment response, tumor growth, and drug concentrations, significantly improved overall survival. Our findings in a mouse model of triple-negative breast cancer provide compelling evidence that chemotherapy can be personalized and optimized through algorithm-assisted therapy design.

## Introduction

Chemotherapy remains one of the most widely used cancer treatments, with nearly 60% of all cancer patients receiving some form of it during their illness^1^. Despite the rise of targeted and biological therapies, many patients lack actionable mutations, leaving chemotherapy as their only treatment option. Interestingly, while novel compounds are developed and new treatment strategies are established, current chemotherapy protocols largely follow a “one size fits all” approach, administering Maximum Tolerable Doses (MTD) on fixed schedules. This approach frequently drives drug resistance or treatment discontinuation due to toxicity. Although dose and scheduling were shown decades ago to be critical for therapeutic efficacy^2^, MTD remains the standard dosing strategy^3^. Metronomic chemotherapy was introduced to address these limitations by using significantly lower doses with more frequent administration and minimal drug free time intervals^4^. The aim was to maintain an effective yet lower plasma concentration of the drug to reduce side effects. However, despite initial promise, metronomic chemotherapy has shown only moderate benefits in selected cancer patients compared to MTD-based therapy in phase II clinical studies^5^.

Breast cancer is the most common malignancy in women and its treatment is tailored to the molecular subtype and generally involves a combination of locoregional approaches (surgery and radiotherapy) and systemic therapies that include endocrine therapy for hormone receptor–positive disease, chemotherapy, anti-HER2 agents for HER2-positive tumors, poly(ADP-ribose) polymerase (PARP) inhibitors for BRCA mutation carriers, and, more recently, immunotherapy. If pathological complete response (pCR) is not achieved, subsequent escalation of systemic therapy is recommended^6^. Nevertheless, roughly 30% of women with early-stage breast cancer eventually progress to metastatic disease, where treatment options remain limited^7^. Anthracycline–taxane combinations are among the most effective regimens for reducing breast cancer recurrence and mortality, with the greatest benefit observed at higher cumulative doses^8^.Unlike other breast cancer subtypes, which have targeted and biological treatment options, cytotoxic chemotherapy remains the only approved treatment for early triple negative breast cancer (TNBC) and is often administered in a neoadjuvant setting. In TNBC, both pre-clinical and clinical studies have shown limited benefits of metronomic chemotherapy compared to MTD dosing^9^. For example, using a metastatic TNBC mouse model, Di Desidero et al. showed that metronomic topotecan improved survival over MTD topotecan only when combined with pazopanib; as monotherapy, it was inferior^10^. Even when metronomic chemotherapy proved to be more efficient, patient selection and treatment protocols were highly heterogenous, and, more importantly, the optimal metronomic dosing and administration scheduling have never been established^11^.

Personalizing cancer therapy is crucial to overcoming chemotherapy resistance. Therefore, in vivo tumor models that closely mimic key characteristics of cancer in human patients, such as the development and impact of drug resistance, are essential. A widely used example is the murine Brca1^-/-^;p53^-/-^ genetically engineered model of hereditary TNBC, which closely resembles human breast cancer^12^. Similar to most human malignancies, these tumors initially respond to treatment but eventually develop resistance to docetaxel, doxorubicin, topotecan, olaparib, or PLD, due to increased expression of the Abcb1 or Abcg2 genes^13–15^. TNBC, the most deadly subtype of human breast cancer, is marked by early relapse and poor overall survival rates, driven by the emergence of diverse and increasingly complex drug resistance mechanisms, including several recently uncovered pathways^16^.

In this study we employ an algorithm-assisted therapy design (AATD) to evaluate the efficacy of individualized protocols in the treatment of murine mammary tumors with PLD. By identifying measurable tumor- and systemic parameters, we establish personalized treatment plans and demonstrate that individualized chemotherapy significantly improves overall survival in a genetically engineered TNBC mouse model compared to conventional tumor-triggered treatment protocols. Furthermore, we showcase the effectiveness of two distinct AATD strategies: one designed to maintain a stable, low, continuous plasma concentration of doxorubicin, and the other aimed at achieving complete tumor eradication.

## Results

### Tumor-triggered treatment protocol leads to the emergence of drug resistance

**Fig. 1:**
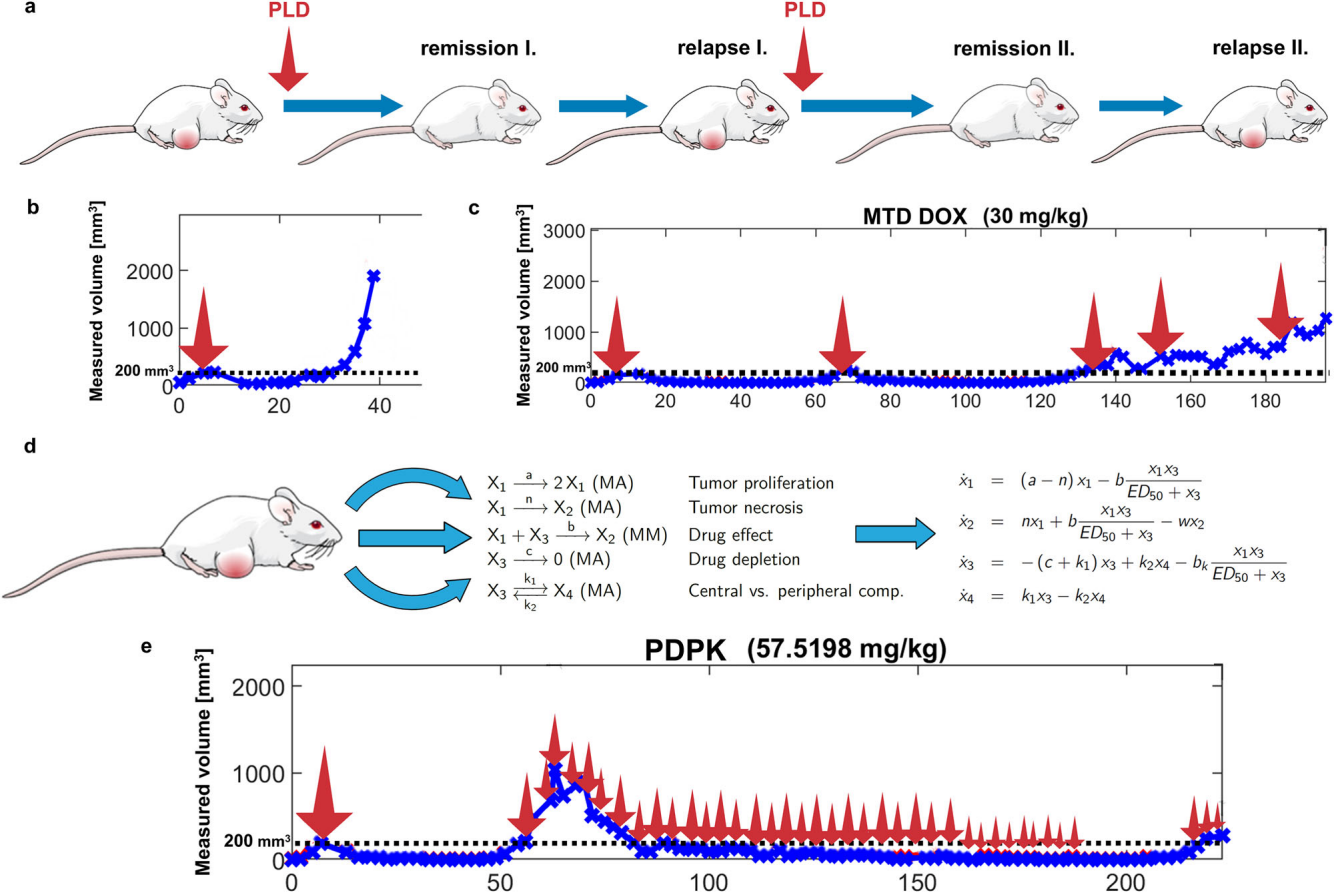
Tumor-triggered vs AATD treatment. **a** Schematic diagram of tumor-triggered therapy. Treatment with MTD starts when tumors reach ~200 mm^3^. Treatments with MTD are repeated at each relapse, when the tumor volume reaches ~200 mm^3^. **b** Representative growth of a tumor with a single treatment. While the treatment with a single MTD of PLD leads to a prolonged remission, tumors universally relapse. **c** Representative growth of a tumor during tumor-triggered therapy. Initial treatments induce significant tumor response leading to extended remission periods (days 10 to 70 and 70 to 130). However, over time, cancer cells gradually lose sensitivity to PLD, and treatment efficacy declines. Beyond day 130, only a partial response is observed, merely slowing tumor growth rather than inducing remission. The arrows indicate the timing and dosage of the treatment, with arrow size proportional to the administered dose. The cumulative dose is indicated in brackets. **d** Ordinary differential equations used in AATD predict tumor behavior, based on key biological parameters, which can be derived from simple data such as tumor volume and PLD blood concentrations. **e** Representative growth kinetics of a tumor under an AATD regimen. During the initial treatment with MTD of PLD (first arrow) the response is monitored through tumor volume measurements and the required biological parameters are calculated using the equations shown in **d**. Upon tumor relapse, the individualized AATD protocol is applied. The arrows indicate the timing and dosage of the treatment, with arrow size proportional to the administered dose. The cumulative dose is indicated in brackets.

Tumor-triggered therapy, frequently applied in the Brca1^-/-^;p53^-/-^ tumor model, involves administering chemotherapeutics at the MTD (Fig. 1a)^17–21^. As frequently observed in the clinical setting, initial treatment induces remission, leaving either no detectable malignancy or only a minimal residual tumor burden^22^. However, over time (~30 to 70 days, depending on the treatment), tumors invariably relapse (Fig. 1b) and MTD treatment is re-administered once the tumor regrows to a defined size. In the case of PLD, repeated use of MTD at each relapse can significantly increase survival, but eventually the response is subdued with the emergence of drug resistance (Fig. 1c). Unlike tumor-triggered therapy, in which MTD treatment is initiated upon tumor relapse, AATD seeks to predict tumor behavior using differential equations based on individual biological parameters. These parameters describe cancer cell proliferation rate, tumor necrosis, the washout rate of dead tumor cells, drug efficacy, drug depletion kinetics, and drug distribution across different compartments (Fig. 1d). To ensure that AATD aligns with clinical oncology practices, most parameters were derived from a limited set of measurements. Tumor proliferation rate, washout rate, rate of necrosis and the effect of treatment were estimated through continuous tumor size monitoring and growth kinetics analysis^23^. Additionally, preliminary assessment of PLD pharmacokinetics allowed the estimation of drug levels in the blood in any given time. We applied two AATD strategies: one aimed at the complete eradication of tumors (Model Predictive Control, MPC), while the other sought to maintain a stable concentration of doxorubicin in the circulation (PDPK). The individualized therapy regimens determined both the timing and dosage of PLD administration (Fig. 1e).

### Tumor growth can be controlled through AATD

**Fig. 2:**
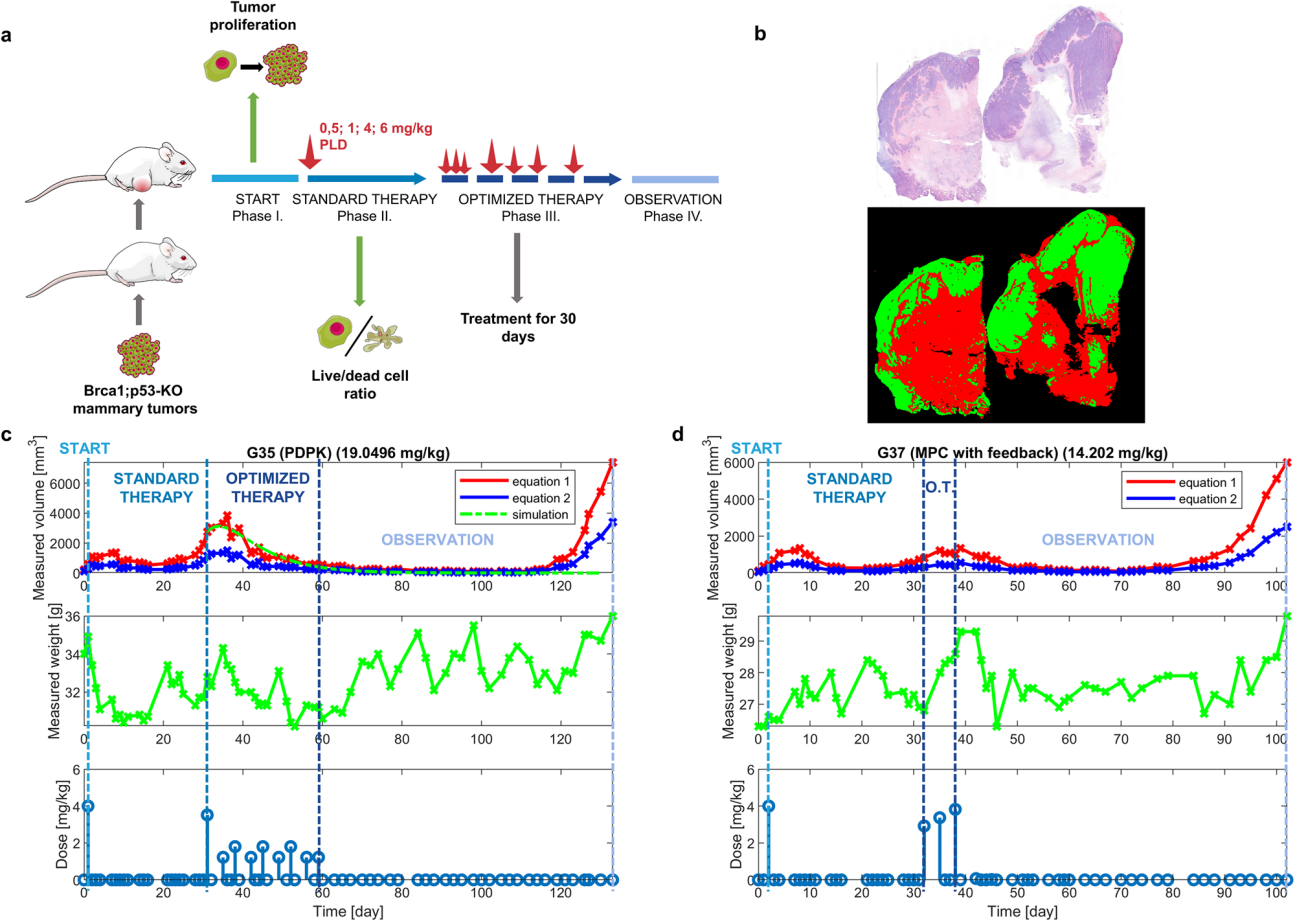
Workflow and results of the pilot AATD experiment. **a** Overview of the experimental scheme. Tumor pieces obtained from Brca1;p53-KO mammary tumors were orthotopically transplanted into wild-type FVB/N mice, and the tumor volumes were monitored every second day throughout the experiment (Phase I). Phase II commenced when tumors reached approximately 200 mm³, at which point a standard chemotherapy protocol was initiated with four different PLD doses (0.5, 1, 4, and 6 mg/kg). One tumor per each group was excised for the determination of the live/dead cell ratio. The biological parameters collected during Phases I and II were utilized to develop individualized treatment protocols for each mouse during Phase III. Upon relapsing to 200 mm³ following Phase II, mice received individualized AATD-based therapy. Treatment continued for 30 days, and survival was monitored until tumors reached ~2000 mm³ in Phase IV. **b** The live/dead cell ratio was determined through image analysis, with green areas indicating live tumor cells and red areas indicating dead cells. Experimental results illustrating the AATD-based therapy using either the PDPK (**c)** or the MPC (**d**) protocol, with cumulative doses (in brackets) administered during the optimized therapy phase. The upper plots show changes of the tumor volume in time, calculated with two equations: Eq. 1 (blue) and Eq. 2 (red) as described in Methods, along with the model-based prediction (green) during the algorithm-assisted treatment phase, using fixed parameters estimated from the previous segment (standard therapy). The simulation starts from the beginning of the optimized therapy to reflect the use-case of model-driven treatment planning.The middle plots show changes of the body mass of the mouse in time. The lower plots shows the administered dose.

First, an experimental workflow was established to provide a framework for the testing of the efficacy of the AATD protocols, and to identify the biological parameters that can influence the individualized therapy design (Fig. 2a). In Phase I (START), Brca1^-/-^;p53^-/-^ tumors were orthotopically transplanted into wild-type FVB/N mice, and the tumor growth was closely monitored to determine tumor cell proliferation, an essential input parameter for the algorithm. When tumors reached 200 mm^3^, a single, fixed dose PLD treatment was applied, simulating conventional chemotherapy. This phase (STANDARD THERAPY, Phase II) allowed for the assessment of tumor response to different doses of PLD through frequent tumor volume measurements. Additionally, in this phase, plasma PLD levels were investigated in every group at day 8. No reduction of tumor volume was observed in mice treated with 0.5 or 1 mg/kg doses of PLD, leading to aggressive tumor growth. However, detailed analysis revealed that both the 0.5 and 1 mg/kg doses slightly altered the tumor growth kinetics compared to the untreated control (Supplementary Fig. 1a,b). In Phase II, one mouse from each treatment group (0.5, 1, 4 and 6 mg/kg) was sacrificed, and the tumor was removed for live/dead cell ratio analysis using image processing (Fig. 2b). The data from the first two phases were then used to develop individualized treatment protocols for each mouse for a 30-day period (OPTIMIZED THERAPY, Phase III).

The PDPK and MPC methods are fundamentally different: while the PDPK algorithm creates a 30-day treatment schedule based on initially collected data, MPC adjusts the treatment daily, considering changes in tumor size before the calculation of each dose. After 30 days of the individualized treatment schedule, the OBSERVATION (IV) phase began to assess the efficacy of the therapies in each group.

The study was designed to identify the key biological parameters for AATD and the minimal set of measurements to derive them. As cancer cell proliferation, necrosis, dead cancer cell washout rate, response to treatment, and pharmacokinetics predict overall tumor growth (Fig. 1d), in our algorithm, we estimated these parameters using continuous tumor volume measurements. Tumor growth kinetics provided sufficient data to calculate proliferation, necrosis, and washout rates, to assess PLD efficacy by comparing tumor size before and after treatment, and to finetune pharmacokinetic parameters^24,25^. To estimate the pharmacokinetics of PLD, blood levels were measured after treatment in a preliminary experiment using healthy, non-tumor-bearing mice, as described in the Methods section. The estimated parameters were then applied as initial values for model identification, during which individual parameter values were refined. These two datasets provided all the necessary starting values for AATD.

The PDPK approach used relatively small, continuous doses to maintain a constant plasma doxorubicin concentration, as shown by a representative result (Fig. 2c). The pre-generated treatment schedule successfully induced remission with no therapy-altering side effects during Phases III and IV. The MPC protocol produced a similar effect, but required fewer injections during the OPTIMIZED THERAPY phase (Fig. 2d, Supplementary Fig. 2).

Despite confirming efficient tumor control with significant relapse free survival times, all tumors relapsed after AATD treatments, indicating that AATD schedules must be maintained for a prolonged period to reach their full potential (Supplementary Fig. 3).

### AATD increases overall survival compared to the tumor-triggered treatment protocol and only rarely induces drug resistance

**Fig. 3:**
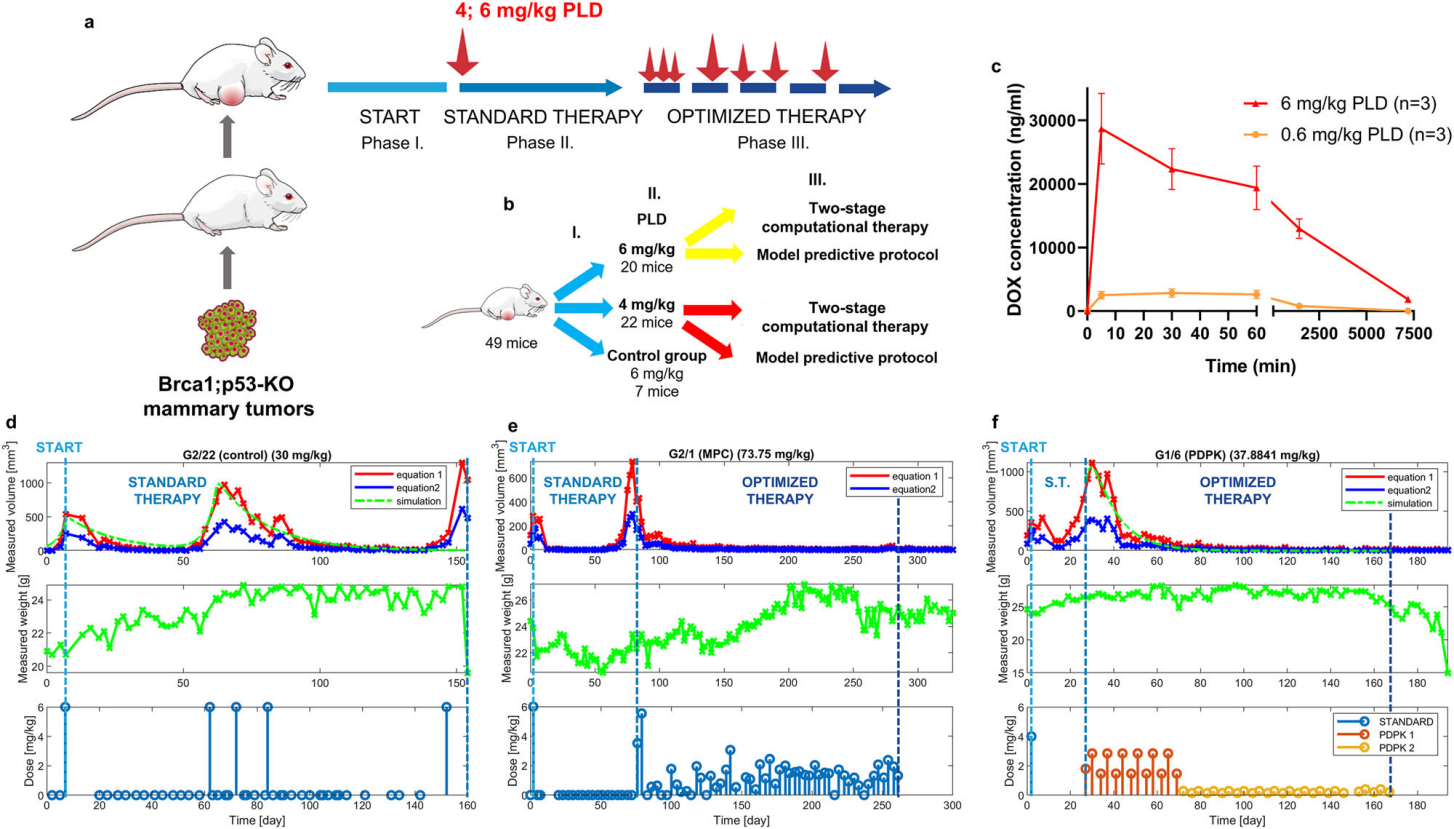
Revised workflow and results of the improved AATD treatment protocols. **a** Following of the engraftment of tumor pieces (Phase I), standard therapy was initiated with 6 mg/kg PLD for the control group or with 4 or 6 mg/kg for AATD (Phase II). Individualized therapy (Phase III) was based on two different, individually generated protocols (PDPK and MPC), in which the animals were treated until the emergence of drug resistance or full tumor control. **b** Experimental design and grouping of the animals. **c** Pharmacokinetics of 0.6 and 6 mg/kg PLD in healthy mice. Serum doxorubicin concentrations were measured in three mice per dose. **d** Representative results obtained from the tumor-triggered MTD treated group simulating conventional chemotherapy (mouse G2/22). The three plots show the changes in tumor growth (upper), in body weight (middle) and the administered PLD doses (lower). Treatments were only applied when the tumor volume reached or were above the 200 mm^3^ threshold. **e** Representative result from the MPC protocol group (mouse G2/1). **f** Representative result from the PDPK protocol group (mouse G1/6). The green curve represents model-based prediction during the algorithm-assisted treatment phase, using fixed parameters estimated from the previous segment (Standard Therapy). The simulation starts from the beginning of the optimized therapy to reflect the use-case of model-driven treatment planning.

Based on the experiences of the pilot experiment, the AATD workflow was revised (Fig. 3a). The OBSERVATION phase was removed, and the tumor-triggered control and AATD treatments were applied until the tumor volume reached 2000 mm^3^, or the mice had to be sacrificed due to severe side effects of the treatment. Additionally, the initial 0.5 and 1 mg/kg doses of PLD in the STANDARD THERAPY phase were eliminated as they failed to reduce the tumor volume. Accurate estimation of plasma drug proved critical for the success of AATD, prompting a detailed pharmacokinetic analysis of 0.6 and 6 mg/kg PLD treatment in tumor-free healthy mice (Fig. 3b). Following the injection of 6 mg/kg PLD, the plasma concentration of free doxorubicin sharply and continuously decreased, whereas the 0.6 mg/kg dose reached an almost constant plasma concentration within the same timeframe. Following the establishment of the new experimental plan and the refined pharmacokinetic profile, the study proceeded with further investigations. 49 tumor-engrafted wild-type FVB/N mice were randomly grouped in 3 groups receiving treatment based on tumor-triggered, PDPK or MPC protocols (Fig. 3b).

In the tumor-triggered MTD treatment group, despite a significant improvement in overall survival, most therapies ultimately failed due to the development of drug resistance, as shown in the representative figure (Fig. 3d, Supplementary Fig. 4a). It is worth noting that the mathematical model does not incorporate an explicit resistance mechanism; instead, relapse can emerge from tumor regrowth following drug decay, when the growth rate exceeds the cell-killing rate. In contrast, when the algorithm-assisted therapy achieves effective tumor control, no relapse is observed within the simulation timeframe, as the parameters reflect sustained suppression. The MPC algorithm resulted in a more aggressive treatment, prescribing a wide range of doses throughout the therapy to tackle tumor growth (Fig. 3e, Supplementary Fig. 4b). The PDPK approach utilized relatively small doses to gain control over the tumor or, at least, slow down its growth (Fig. 3f, Supplementary Fig. 4c). Detailed results for all tumors and treatments are provided in the Supplement.

**Fig. 4:**
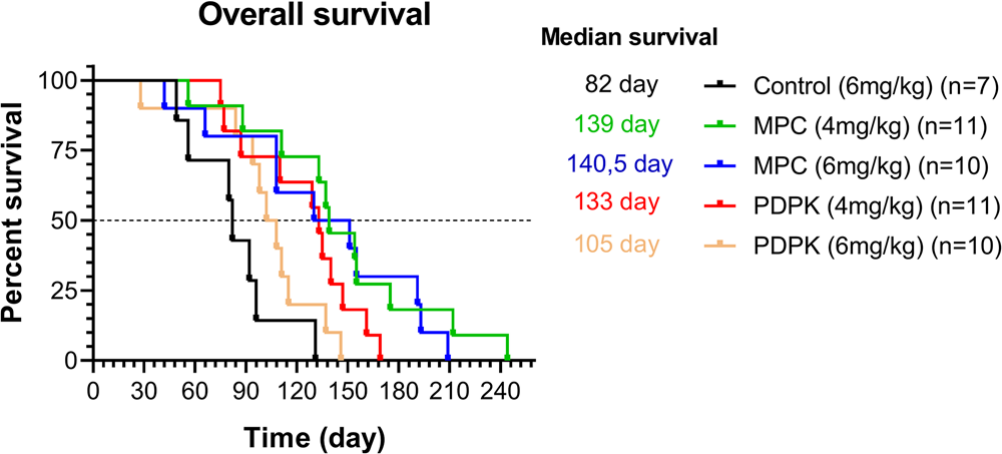
Overall survival of different treatment groups. The Kaplan-Meier curves demonstrate the survival differences between the control (black) and MPC (green and blue) and PDPK (red and yellow) treatment groups. The analysis was performed using the GraphPad Prism version 8.0.0 for Windows.

Ultimately, AATD treatment schedules significantly improved overall survival compared to tumor-triggered MTD therapy (Fig. 4). All AATD protocols outperformed the tumor-triggered therapy (‘control’), with three out of four showing statistically significantly differences [MPC 4 mg/kg (HR: 0.12, 95% CI: 0.04–0.35, *p* = 0.0002), MPC 6 mg/kg (HR: 0.15, 95% CI: 0.05–0.45, *p* = 0.0008), PDPK 4 mg/kg (HR: 0.25, 95% CI: 0.09–0.69, *p* = 0.0076)]. PDPK 6 mg/kg also improved survival, though the difference was not significant (HR: 0.48, 95% CI: 0.18–1.29, *p* = 0.1442).

## Discussion

Chemotherapy, used to treat millions of patients annually, remains a mainstay of cancer treatment. However, research aimed at improving chemotherapy has been limited (Füredi et al.,^45^), largely due to the long-standing dogma that chemotherapy cannot be personalized. Metronomic chemotherapy was developed based on the concept that anticancer agents can selectively target premature, constantly proliferating endothelial cells in the tumor vasculature due to the almost permanent need for angiogenesis in its microenvironment^26^. This concept was later validated in animal studies, where mice with subcutaneous tumors responded to frequently administered low dose chemotherapy, even when the same tumors had become resistant to the drug given under conventional MTD schedules^2,27^. Metronomic chemotherapy has generated great interest over the last decade, however the results of clinical studies have been heterogenous, ranging from significant improvement to no benefit in overall survival^28–31^.

One of the challenges in translating metronomic chemotherapy to clinical practice is the lack of established low-dose regimens that remain effective while maintaining a favorable toxicity profile for chronic treatment^31^. In this work, we present a fundamentally different approach to personalizing chemotherapy, combining the benefits of MTD and metronomic chemotherapy to enhance the efficacy of conventional chemotherapy.

Ordinary differential equations were used to model and predict tumor behavior based on a limited set of biological parameters, and two different treatment approaches were tested to improve survival of tumor-bearing mice. Algorithm-assisted therapy design controlled the dosing and timing of PLD administration to maximize therapeutic effect. We optimized the experimental workflow to test AATD in a mouse model of TNBC and to identify the biological parameters most critical for effective therapy planning and treatment outcome. Tumor size, tumor response and plasma drug concentration were confirmed to be the key parameters that must be precisely measured for successful AATD.

AATD outperformed conventional tumor-triggered therapy using MTD, significantly improving overall survival. To our knowledge, only one similar system is currently under evaluation: CURATE.AI, an artificial intelligence-driven phenotypic personalized medicine platform designed to identify optimal dosing schedules for patients^32,33^. While AATD and CURATE.AI share similarities, CURATE.AI relies on blood-based biomarkers (e.g., CEA or CA19-9) to estimate treatment response and calculate drug doses that can decrease the levels of these markers. Although this strategy can be effective in some cases, many solid tumors do not secrete reliable biomarkers, limiting its applicability.

The clinical translation of AATD is feasible, as the required biological parameters can be measured using existing technologies. Tumor size and treatment response can be monitored via medical imaging techniques such as ultrasound, X-ray, CT, MRI, PET, or by using reliable blood biomarkers. Therapeutic drug monitoring (TDM) in the patient’s circulation can be performed using high-performance liquid chromatography (HPLC), mass spectrometry (MS) or, in some rare cases, immunoassays. Our recent work explored the optimal frequency for monitoring individual biological parameters to minimize the number of measurements, which could aid in optimizing AATD for clinical application^34^.

The optimal clinical protocol of AATD would involve the following steps: 1. The patient is diagnosed with cancer, and standard chemotherapy is initiated. 2. During standard therapy, key biological parameters, such as the tumor’s response to chemotherapy and the patient-specific pharmacokinetics of the drugs, are determined. 3. Using the obtained parameters, AATD is performed to individually optimize treatment dosing and scheduling according to the target goals. Although current technologies could enable AATD, several challenges may hinder its clinical translation. Personalized dosing schedules could be demanding for both patients and doctors, as optimized therapies may require daily treatment, while conventional protocols typically involve a single dose of chemotherapy every three weeks. Additionally, frequent but low-dose treatments may be economically impractical due to drug constraints imposed by packaging, as once a vial is opened, the remaining drug cannot be reused. Issues with frequent dosing could be potentially addressed with autonomous wearable drug delivery devices, however these systems have only recently emerged for cancer treatment purposes^35,36^. Despite these challenges, with procedural optimization, AATD has the potential to be successfully integrated into clinical practice. Overall, despite the challenges, AATD represents a novel, clinically feasible approach to individualized treatment planning, using readily accessible biological parameters to enhance the effectiveness of conventional chemotherapy.

## Methods

### Dose optimization based on two-stage computational therapy (PDPK)

The AATD methods are based on a mathematical model that consists of ordinary differential equations to describe how the tumor volume evolves in time, taking into consideration the effect of the treatment, along with the drug concentration in the patient^25,37^. The model can be used to create a digital twin of the mouse, simulate the effect of the drug in advance, and choose the dose predicted to achieve optimal therapeutic outcomes. The differential equations were derived based on physiological considerations; therefore, the model parameters characterize physiological phenomena and serve as inputs for the two-stage computational therapy approach, rather than being used directly for simulation.

The mathematical model can also be exploited to influence tumor volume evolution while accounting for several constraints (e.g., dose limits, cumulative dose, injection frequency)^38^. In the frame of the two-stage computational therapy, we used the pharmacokinetic model of pegylated liposomal doxorubicin to calculate the minimal doses required to maintain drug levels above a specified threshold, referred to as the minimal inhibitory concentration (MIC) following Jacobs^39^.

The MIC can be calculated according to two strategies, depending on the tumor stage^38^. Initially, when the tumor volume is large, MIC is calculated as the drug level that achieved a significant therapeutic effect while balancing efficacy and toxicity; set to 99% effectivenes in the pilot study. This calculation relies on the pharmacodynamic model using the median effective dose parameter described in Supplementary Material 1, and typically results in a smaller dose than the MTD (for a detailed rationale, see Kovács et al.^38^). If the tumor volume is under a specified threshold (20 mm^3^ in the experiment) for two weeks, MIC is determined using the tumor dynamics model to identify the minimal dose that prevents tumor regrowth. The detailed mathematical formulation and strategies are described in Supplementary Material 1. Given the combined use of the pharmacodynamic (PD) and pharmacokinetic (PK) model, we refer to this algorithm as PDPK, emphasizing its physiological basis, as opposed to the model predictive control approach, which, methodologically, can use any mathematical model regardless of its physiological interpretability.

The mathematical model was also used to simulate tumor growth based on parameters estimated prior to the optimized therapy phase in Figs. 2c and 3f. However, in Fig. 3.d., where simulation curve starts from the beginning of treatment, the model was not used for therapy design, and the simulation illustrates the retrospective fit of tumor dynamics using fixed parameters. In contrast, for algorithm-assisted treatments (e.g., Figs. 2c and 3f), simulation starts from the onset of the optimized therapy phase to reflect model-based prediction during treatment.

In Fig. 3d, the entire course of treatment is simulated because no AATD was applied in this group. The relapse observed around day 50 emerges naturally as the drug is eliminated from the system and the tumor resumes growth before the next treatment, while the model parameters remain fixed. By the end of the treatment period, the high cumulative dose has markedly reduced the tumor volume, resulting in slower regrowth. Consequently, by the time the tumor could begin to relapse again, another treatment is administered around day 150, effectively preventing a second visible relapse in the simulation as opposed to the measurement, which shows tumor relapse due to developed resistance not captured by the mathematical model.

### Nonlinear Model Predictive Control (MPC)

MPC is an optimal control strategy extensively described in Kovács et al.^23^, which uses predictions on the evolution of the tumor volume to guide therapy. The algorithm begins by measuring tumor volume, followed by the definition of a prediction horizon, a fixed time interval over which tumor growth is forecasted. Within this interval, a fixed number of drug administrations is scheduled, with constant timing but adjustable dose magnitudes.

Using prior administration data and measurements, an online parameter identification method updates the mathematical model parameters. The model then simulates the effects of potential doses over the prediction horizon, and an optimization algorithm selects the dose magnitudes that minimize a cost function balancing the squared tumor volume against the squared sum of the administered drug doses. Further methodological details are provided in Supplementary Material 1.

### Chemotherapy compound

Pegylated liposomal doxorubicin (PLD, Caelyx®, Janssen) was chosen for the AATD experiments based on the significantly higher efficiency in a mouse model of TNBC, prolonged circulation time and greater plasma concentration levels compared to conventional doxorubicin^13^. The compound was acquired directly from the manufacturer.

### Animal experiments

Tumor tissue samples (approximately 1-2 mm in diameter) from Brca1^−/−^;p53^−/−^ FVB/N mice (kindly provided by Sven Rottenberg, NKI, Netherlands and University of Bern) were transplanted orthotopically into the mammary fat pads of 46 (for the pilot experiment) and 49 (for the main study) female wild-type FVB/N mice (Harlan) under anesthesia (20 mg/kg zolazepam, 12.5 mg/kg xylazine, 3 mg/kg butorphanol, 20 mg/kg tiletamine). The growth of the transplanted tumors was monitored on a regular basis, with caliper measurements taken three times a week after the tumors became palpable. Tumor volume was calculated using the formulas

1 $${\mathrm{V}}={\mathrm{length}}\times ({{\mathrm{width}}}^{2}/2)$$

2 $${\mathrm{V}}=\pi /3\,{({\mathrm{length}}\times {\mathrm{width}})}^{(3/2)}$$

which we will refer to as Eqs. 1 and 2, respectively. Treatment started when tumor volume reached approximately 200 mm^3^ based on Eq. 1. Animals were sacrificed using cervical dislocation when the tumor volume reached ~2000 mm^3^, calculated using Eq. 1.

All animal experiments were conducted in compliance with ethical guidelines established by the Hungarian Animal Health and Animal Welfare Directorate, which are in line with 2010/63/EU of the European Parliament and the Council of the European Union on the protection of animals used for scientific purposes. All surgical and treatment procedures were carried out in accordance with “Guiding Principles for the Care and Use of Animals” based on the Helsinki declaration, and they were approved by the ethical committee of the National Institute of Oncology in Budapest, Hungary, ensuring that the animals were treated humanely. The permission licenses were PEI/001/1738-3/2015 and PE/EA/1461-7/2020.

### Pilot experiment for AATD treatments

To evaluate drug response and feasibility of AATD, a pilot experiment was performed. Initially, 0.5, 1, 4 and 6 mg/kg PLD were administered intravenously through the tail vein and a mouse-specific biological parameter, the tumor volume, was calculated using Eq. 2 for every mouse, while plasma drug concentrations for each dose were determined by mass spectrometry 8 days after administration in 3 mice/dose.

Initially, the mice received a fixed dose treatment. Individual therapy was started when the tumors relapsed and their volume reached 200 mm^3^ again. After that, the mice were randomly divided into new groups based on the AATD strategy (PDPK or MPC). The tumor volume measurements up to the start of the individualized therapy were used to carry out parametric identification of the mathematical model of the tumor; the unique parameters of the mice were calculated using mixed-effect models^19–21^. The AATD strategies used these unique parameters to generate personalized treatment. The animals were treated continuously according to individually generated protocols for 30 days and no additional doses were administered after. Survival was monitored until each tumor volume reached 2000 mm^3^ when the animals were euthanized.

### Evaluation of live and dead tumor tissue

Following removal, tumor tissues were washed in ice-cold PBS and fixed in 4% formaldehyde solution for 48 hr. The tissues were then dehydrated, and sectioned into 5 μm thick slices, stained with hematoxylin and eosin, and mounted using ProLong Gold (Life Technologies). Images of the hematoxylin- and eosin-stained tissue samples were captured with a Pannoramic 1000 whole-slide scanner (3DHISTECH) at 20× objective magnification, yielding a resolution of 0.25 μm/pixel. To facilitate automated image processing, the digitized samples were resized by a factor of 16, reducing the image size. At this reduced resolution, individual cells were no longer discernible; however, distinct tissue regions remained clearly distinguishable.

A two-phased automated image processing workflow was employed to calculate the ratio of necrotic tissue. In the first phase, pixel classification was performed on the resized images using Ilastik^40^. Each pixel was assigned with one of three predefined labels: viable tissue, necrotic tissue, or background. A random decision forest classifier was trained using a separate set of histologist-annotated samples to ensure accurate labeling. Features such as color, intensity, edges, and texture from the input images were utilized to predict the assigned labels. The resulting prediction probability maps were then imported into a custom-built CellProfiler^41^ processing pipeline. A prediction acceptance threshold of 50% was applied to each category. To eliminate small artifacts in the resulting images, median filtering was applied using a 5×5 sampling window.

The necrotic ratio (η_*i*_) was calculated for each image *i* using the formula:

3 $${\eta }_{i}={N}_{i}/({N}_{i}+{V}_{i})$$

where *N*_*i*_ and *V*_*i*_ represent the number of pixels classified as necrotic and viable tissue, respectively. The lower limit of η is zero, indicating the absence of necrotic tissue within the image, which suggests that the treatment was ineffective. Conversely, values of η approaching one signify that the tissue is predominantly necrotic, reflecting 100% treatment efficiency. Thus, η serves as a quantitative measure of cytotoxicity and treatment success.

### Measurement of plasma doxorubicin levels after PLD treatment

To measure plasma concentrations of PLD, FVB/N mice were treated with a single dose of PLD of 0.5, 1, 4, and 6 mg/kg and blood samples were taken 8 days after treatment. To assess pharmacokinetics of PLD FVB/N mice were given a single intravenous dose of PLD of 0.6 or 6 mg/kg. Blood samples were collected from the mice before administration and after at 5, 30, 60, 1440, and 7200 minutes by cardiac puncture of euthanized animals. Plasma was separated from the blood cells by centrifuging at a speed of 4000 rpm for 15 minutes at 4°C. After separation, the samples were subjected to acetonitrile protein precipitation (10x dilution). The LC-MS/MS analysis was conducted using a QTRAP 6500 triple quadruple linear ion trap mass spectrometer from AB Sciex (California, USA), which was equipped with a Turbo V Source for electrospray mode and a Perkin Elmer Series 200 micro-LC system (Massachusetts, USA). These measurements were used to identify the parameters $c$, ${k}_{1}$, and ${k}_{2}$ of the pharmacokinetic submodel of the mathematical model using mixed-effect modeling (see the Supplementary Material).

### Testing redefined AATD protocols

Experimental AATD workflow was redefined based on the experiences obtained during the pilot study. The standard, fixed dose therapy phase was initiated when the tumor volume reached 200 mm^3^ only with 4 and 6 mg/kg doses of PLD intravenously, and mouse-specific biological parameters were collected as before, however the ratio of live and dead cancer cells was not monitored anymore and a complete pharmacokinetic profile for plasma PLD concentration was established using 0.6 and 6 mg/kg doses of PLD measured by mass spectrometry.

Individualized therapy was started when the tumor volume increased to 200 mm^3^ again after the first, fixed dose 4 or 6 mg/kg treatment, then the mice were randomly divided into new groups based on the AATD strategy (PDPK or MPC). The measurements collected up to this point were used to carry out parametric identification of the mathematical model based on mixed-effect modeling, resulting in a unique parameter set for each mouse.

AATD therapies were compared to the commonly used MTD-based tumor-triggered treatment schedule which administer MTD every time the volume of the tumor relapse to its size at the beginning of the treatment or did not decrease the next 10 days. MTD treatments can be repeated every 10 days.

The animals were treated continuously according to individually generated protocols until each tumor volume reached 2000 mm^3^ or serious side effects were observed when the animals were immediately euthanized.

### Statistical analysis

Calculations were carried out under the R statistical environment version 4.1.0 [R Core Team (2022). R: A language and environment for statistical computing. R Foundation for Statistical Computing, Vienna, Austria. URL https://www.R-project.org/.] using the package survival version 3.4-0^42^. Survival curves were estimated using the non-parametric method of Kaplan and Meier^43^ separately for each group. Equality of the survivals among groups were then tested using the non-parametric log-rank test^44^. A Cox proportional model was fitted to the survival data and the overall differences in survival were tested with likelihood ratio test. Hazard ratios (HR) with 95% confidence intervals and *p*-values are presented for the differences of each group compared to the control group. The proportionality of the hazards was checked with the test of Grambsch and Therneau, which indicated no significant deviation from proportionality (*p* = 0.58). Of note, survival time was calculated starting from the day when the mouse received a dose for the second time to purely assess the effects of AATD. In the rare cases when only a single dose was given, survival was defined as the time between the first treatment to reaching the tumor volume of 2000 mm^3^. The analyses were performed using the GraphPad Prism version 8.0.0 for Windows, GraphPad Software (San Diego, CA, USA) and R version 4.4.2, R Foundation for Statistical Computing (Vienna, Austria).

## Supplementary information

Supplementary Information

## Data Availability

The data generated in this study are included in the main article and its supplementary material. The tumor model and other relevant data are available upon reasonable request from the corresponding authors and subject to ethics approval.
