# Supplementary material for: Algorithm-assisted individualized therapy design improves survival in a mouse model of triple-negative breast cancer
Source: NPJ Precis Oncol. 2026 Jan 19;10:84. doi: 10.1038/s41698-025-01245-5 (PMC12948990; doi:10.1038/s41698-025-01245-5)
Supplement: Supplementary file 1 — Supplementary Information [file 41698_2025_1245_MOESM1_ESM.pdf]

Supplementary Materials

Individualized, algorithm-assisted therapy design significantly increases survival of a mouse model of triple-negative breast cancer

Balázs Gombos, Violetta Léner, Dániel András Drexler, Bence Czakó, Tamás Ferenci, Levente Kovács, Dániel Kiss, Pál Szabó, József Tóvári, Gergely Szakács, András Füredi

Supplementary Material 1

The AATD methods are based on a mathematical tumor growth model comprised of ordinary differential equations. The model describes the tumor dynamics, pharmacodynamics, and pharmacokinetics. The model is general, i.e., it can be used to describe the behaviour of different types of tumors and drugs (these are characterized by the model parameters). The equations are

$$\dot{x}_1 = (a-n)x_1 - b\frac{x_1 x_3}{ED_{50} + x_3} \quad (1)$$

$$\dot{x}_2 = nx_1 - b\frac{x_1 x_3}{ED_{50} + x_3} - wx_2 \quad (2)$$

$$\dot{x}_3 = -(c+k_1)x_3 + k_2 x_4 - b_k\frac{x_1 x_3}{ED_{50} + x_3} \quad (3)$$

$$\dot{x}_4 = k_1 x_3 - k_2 x_4 \quad (4)$$

where x_1 is the time function of the living tumor volume [mm^3], x_2 is the time function of the dead tumor volume [mm^3], x_3 is the time function of the drug level in the central compartment [mg/kg], and x_4 is the time function of the drug level in the peripheral compartment [mg/kg]. The model parameters, which makes the model personalized are the *a* growth rate of the tumor [1/day], *b* maximal rate of the killing effect of the drug[kg/(mg· day)], *c* clearance of the drug [1/day], *n* necrotic rate [1/day], ED_{50} median effective dose of the drug [mg/kg], *w* washout rate of the dead cells [1/day], and k_1, k_2 rates of drug flow from between the central and peripheral compartments[1/day]. The parameter b_k describes the rate at which the drug is consumed while taking effect. However, our earlier works showed that this effect is negligible; therefore, we assume this parameter to be zero[1,2]. The measurable variable of the model is the sum of live and dead tumor volume, i.e., $y = x_1 + x_2$.

The parameters of the model are listed in *Supplementary Table I.* The numerical values of the parameters acquired with mixed-effect modelling after the standard therapy are shown for three mice in *Supplementary Table II.* The table contains the identified parameters based on the measurements carried out during the standard therapy phase. Initially, we assume that the dead tumor cell volume is negligible, i.e., we consider $x_2(0) = 0$ mm^3. Moreover, prior to the first injection, we assume that no drug is present in the body, so $x_3(0) = 0$ mg/kg and $x_4(0) = 0$ mg/kg. The value of x_3 changes when the first injection is administered. This impulsive nature of the differential equation is incorporated into our mixed-effect modeling algorithm. As a result, only $x_1(0)$ is estimated during the identification process.

Let $\boldsymbol{u} = (u_0, u_1, \ldots, u_{K-1})^{\mathrm{T}}$ be the doses of the drug injections given at time instants $t_0, t_1, \ldots, t_{K-1}$, where the doses are added to x_3 at the time instants of the injections. Let

$$w(t) = \frac{\lambda_1 + k_2}{\lambda_1 - \lambda_2} e^{-\lambda_1 t} - \frac{\lambda_2 + k_2}{\lambda_1 - \lambda_2} e^{-\lambda_2 t} \tag{5}$$

be the impulse response of the pharmacokinetic subsystem defined by the last two differential equations of the model, i.e., the time function of the drug level caused by a unit dose injection at time $t = 0$, where

$$\lambda_{1,2} = \frac{-(c + k_1 + k_2) \pm \sqrt{(c + k_1 + k_2) - 4ck_2}}{2}. \tag{6}$$

Let $\mathbf{1} = (1, 1, \ldots, 1)^{\mathrm{T}}$ be a column vector with elements of one and length K, and let Φ be the matrix of impulse responses constructed as

$$\boldsymbol{\Phi} = \{\mathrm{w}(\mathrm{t}_i - \mathrm{t}_{j-1})\}_{i,j} \tag{7}$$

where i, j = 1, 2, . . . , K, and t_K is a time instant after the last injection, i.e., $t_K > t_{K-1}$, representing the last time where we want to keep the drug level in the central compartment over a specified value, i.e. , the goal is to have the drug level over a value denoted by MIC between times 0 and t_K. If we calculate the doses as

$$\boldsymbol{u} = MIC \cdot \boldsymbol{\Phi} \cdot \mathbf{1}, \tag{8}$$

we get the minimal value of the doses to keep the drug level in the central compartment over the limit MIC.

Our two-stage computational therapy, PDPK, is an optimization method that relies on this equation. Since this equation is based on pharmacokinetics (PK), and one of our strategies is based on pharmacodynamics (PD), we use the short name PDPK for our algorithm to emphasize the physiological intuition. In the optimization process, the key issue is to specify the value of the MIC. The first strategy is to maximize the effect of the drug based on the pharmacokinetic parameter ED_{50}. By considering $MIC = \kappa\, ED_{50}$, we can tune κ to reach the desired effect. If κ is large, then the therapy is more efficient, but the drug level, thus the toxicity is increased. However, since the parameters ED_{50}, c, k_1, k_2 are tailored to the patient and the drug (which is done in the experiment by parametric identification), this is done in a personalized way. We used $\kappa = 100$ in the phase where the tumor volume was over 20 mm^3 in the pilot experiment, where the ED_{50} parameters were relatively small, resulting in a 99% effect of the drug. This is the first stage of the treatment.

If the tumor was under 20 mm^3 for at least two weeks, we switched to another strategy. This is the second stage of the treatment. We calculated MIC as the minimal value that ensures that the tumor does not grow at any time instants, using

$$\kappa = \frac{a - n}{b - a + n}. \tag{9}$$

This can be derived from the differential equations governing tumor dynamics by specifying that the rate of change of the living tumor volume must be nonpositive[3]. If the parameters of the model are known, this choice will keep the tumor at a low volume. If the parameters of the model change during the treatment (e.g., due to cell mutation), this formulate will not guarantee to keep the tumor in a dormant state. In this case, reidentification of the model parameters is required.

In the redefined experiments, we performed parametric identification based on measurements from standard therapy. The identified parameters were used to capture the core dynamics of the

process and were incorporated into the above formulas to calculate the optimal doses. In some cases, when the tumor did not respond well after the second remission, parametric identification was repeated.
Notably, the PDPK method does not explicitly use the model to simulate the effect of the drug. Instead, it relies on the underlying equations to generate the therapy. The PDPK method is discussed in more detail in Kovács et al.[3].

Another approach to control the tumor volume is the model predictive control (MPC), which relies on the solutions of the differential equations (i.e., predictions of the drug effect based on the mathematical model). Let us formulate the differential equations between the injections at time t_k and t_{k+1} in the compact form

$$\dot{\boldsymbol{x}}_k = f(\boldsymbol{x}_k, \boldsymbol{u}) \tag{10}$$

and let $\boldsymbol{s}_k = \boldsymbol{x}_k(t_k)$ be an instrumental variable which defines the drug level in the central compartment prior to the injection at time t_k, used by a constraint in the upcoming optimization to handle the impulsive nature of the input.
During MPC, we minimize the cost function for segments between the injections t_k and t_{k+1} (for $k = 0,1,\dots,K-1$) as

$$l(\boldsymbol{s}_k, u_k) = \int_{t_k}^{t_{k+1}} \left(\frac{y(t) - y_{ref}}{y_0}\right)^2 + r\left(\frac{u_k}{u_{max}}\right)^2 \mathrm{dt} \tag{11}$$

with y_0 being the tumor volume at the beginning of the treatment, u_{max} being the maximal allowed dose, y_{ref} is the reference tumor volume (the goal is to have the tumor volume under this limit), $y(t)$ is the predicted tumor volume based on the mathematical model, while r is a design parameter used to tune the tradeoff between the speed if the tumor volume decrease and the cumulated dose.
For the whole treatment, we solve the optimization problem

$$\min_{\boldsymbol{s},\boldsymbol{u}} \sum_{k=0}^{K-1} l(\boldsymbol{s}_k, u_k) \tag{12}$$

such that

$$s_k - \overline{\boldsymbol{x}}(t_k) = 0 \tag{13}$$

$$\boldsymbol{s}_{k+1} - \boldsymbol{x}_k(t_{k+1}, s_k, u_k) = 0 \tag{14}$$

$$u_k \in [0, u_{max}]$$

where $x_{3,k}(t_{k+1}, s_k, u_k)$ is the solution of the differential equation at time t_k on the segment $[t_k, t_{k+1}]$ with s_k being the initial condition and u_k the injected dose at time t_k, and $\overline{\boldsymbol{x}}(t_k)$ is the prediction of the moving horizon estimator at time t_k, as discussed in Kovács et al.[4]. The moving horizon estimator is used to track the parameters (a, b, n, w) of the model and uses the result of the identification only as initial values for these parameters, while considers the other parameters constant. The drawback of this approach is that the moving horizon estimator is sensitive to measurement noise, adding propagating the measurement noise to the generated doses. Details about the implementation of the MPC algorithm and the moving horizon estimator can be found in Kovács et al.[4].

Supplementary Table I. Notations, definitions, and dimensions of the parameters of the tumor growth model

Parameter notation	Parameter definition	Parameter dimension
$\boldsymbol{a}$	growth rate of the tumor	1/day
$\boldsymbol{b}$	maximal rate of the killing effect of the drug	1/day
$\boldsymbol{n}$	necrotic rate	1/day
$\boldsymbol{w}$	washout rate of the dead cells	1/day
$\boldsymbol{ED_{50}}$	median effective dose of the drug	mg/kg
$\boldsymbol{b_k}$	modified maximal killing rate	mg/(kg · day · mm^3)
$\boldsymbol{c}$	clearance of the drug	1/day
$\boldsymbol{k_1}$	rates of drug flow from the central to the peripheral compartments	1/day
$\boldsymbol{k_2}$	rates of drug flow from the peripheral to the central compartments	1/day
$\boldsymbol{x_1}$	time function of the volume of the living tumor volume	mm^3
$\boldsymbol{x_2}$	time function of the dead tumor volume	mm^3
$\boldsymbol{x_3}$	time function of the drug level in the central compartment	mg/kg
$\boldsymbol{x_4}$	time function of the drug level in the peripheral compartment	mg/kg

Supplementary Table II. The numerical value of the model parameter acquired from mixed-effect model fit after the standard therapy for mice G3-5, G2-22, and G1-6

Parameter	G1-6	G2-22	G3-5
a [1/day]	0.3494	0.2687	0.3065
b [1/day]	0.9562	2.7602	0.4212
n [1/day]	0.1023	0.0001	0.0031
w [1/day]	0.0895	0.0559	0.1884
ED_{50} [mg/kg]	0.3211	0.6405	0.0113
b_k [mg/(kg ·day · mm^3)]	0	0	0
c [1/day]	0.6831	0.6818	0.1447
k_1[1/day]	28.755	44.3349	26.9802
k_2 [1/day]	182.909	44.3659	11.6601
$x_1(0)$ [mm^3]	44.4682	67.3450	25.6555

Supplementary Figure 1

In the pilot experiment, the mice were treated according to PDPK and MPC protocols with or without feedback. During feedback, all tumor volumes measured up to the day of treatment in the experiment were considered, while without feedback, therapy was designed based only on the tumor volume measured that day.

a

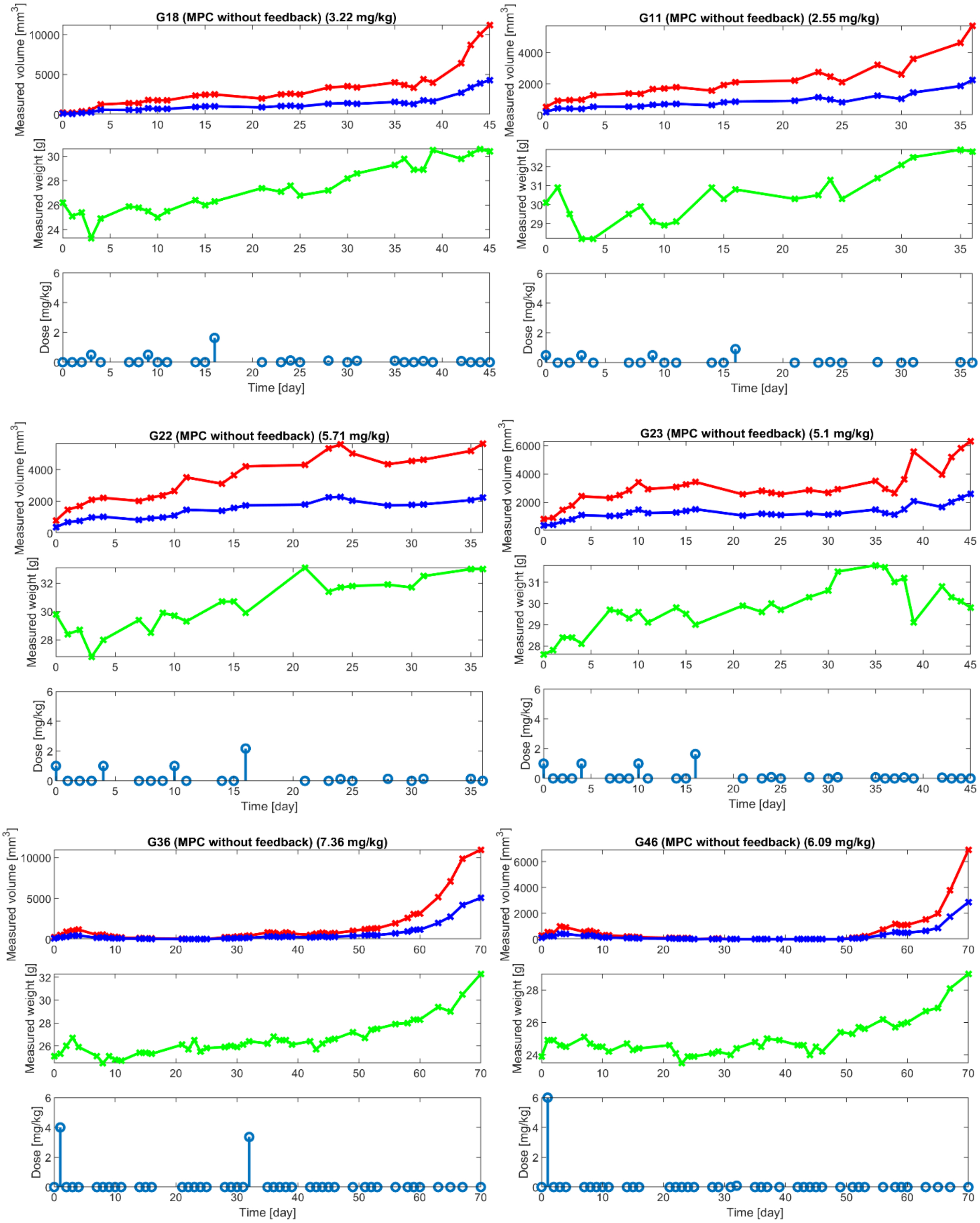

b

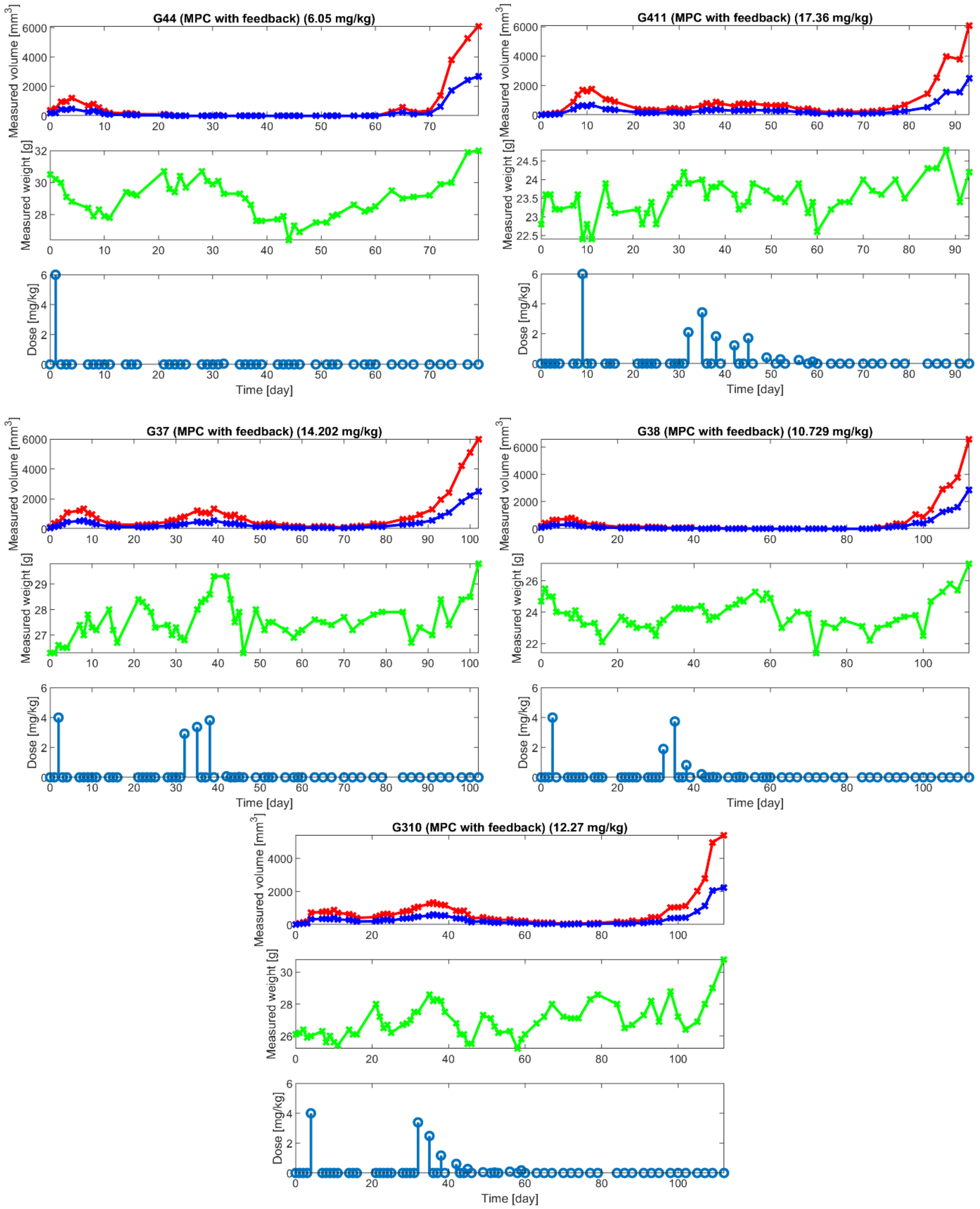

Supplementary Fig 1.: Growth kinetics of MPC tumors in the pilot AATD experiment. Tumor volume was calculated using the V = length × (width^2/2) and V = π/3 (length × width)$^{(3/2)}$ formula (equation 1 and 2).

a Experimental results obtained from the group of MPC without feedback. The first plot shows the evolution of the tumor volume calculated by equation 1 (blue) or equation 2 (red). The second plot shows the evolution of the body mass of the mouse, and the last plot shows the standard and optimized doses. The optimized therapy was based on the MPC.

b The experimental results obtained from the group of MPC with feedback.
The first plot shows the changes in tumor volume in the same manner as describe above.
The second plot shows the evolution of mass of the mice, and the last plot shows the standard and optimized doses. The optimized therapy was based on the MPC.

Supplementary Figure 2

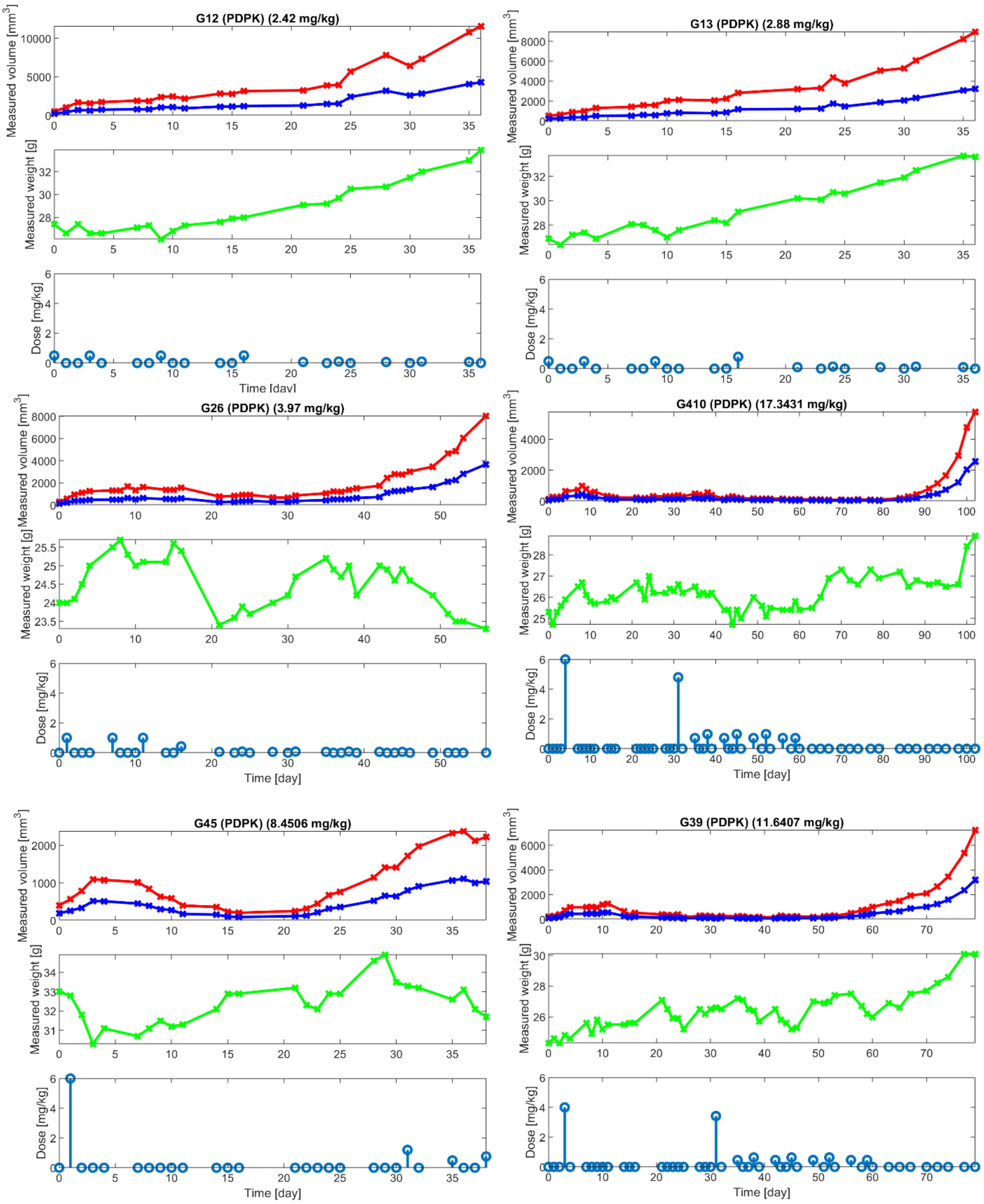

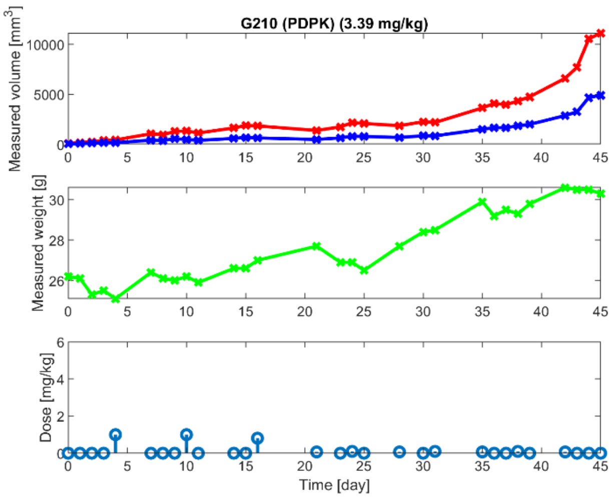

Supplementary Fig 2.: Growth kinetics of PDPK tumors in the pilot AATD experiment. Experimental results obtained from the group of PDPK. The first plot shows the evolution of the tumor volume calculated by equation 1 (blue) or equation 2 (red). The second plot shows the evolution of the body mass of the mouse, and the last plot shows the standard and optimized doses. The optimized therapy was based on the PDPK.

Supplementary Figure 3

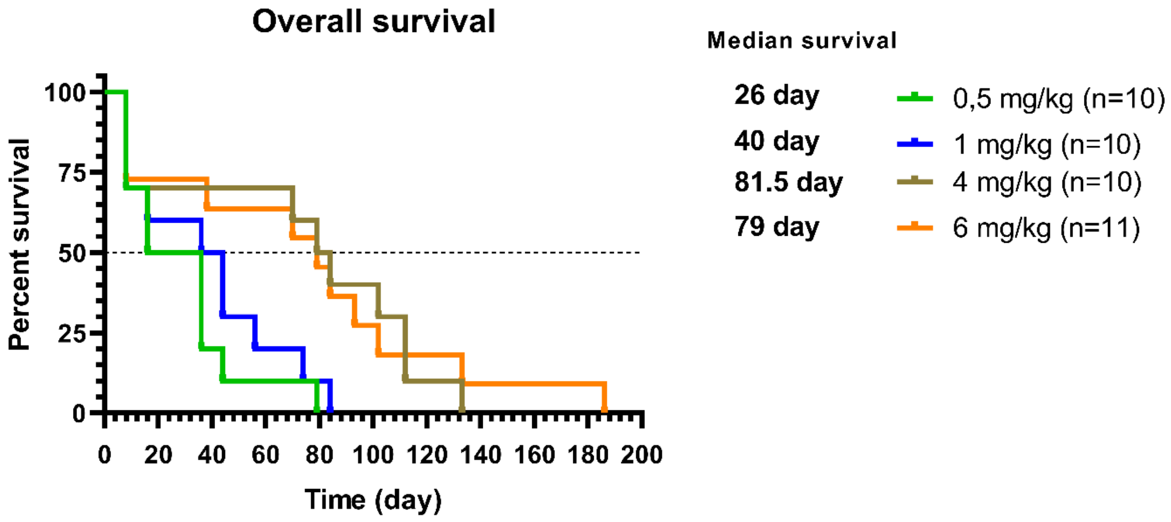

Supplementary Fig 3.: Overall survival of the pilot experiment

Supplementary Figure 4

a

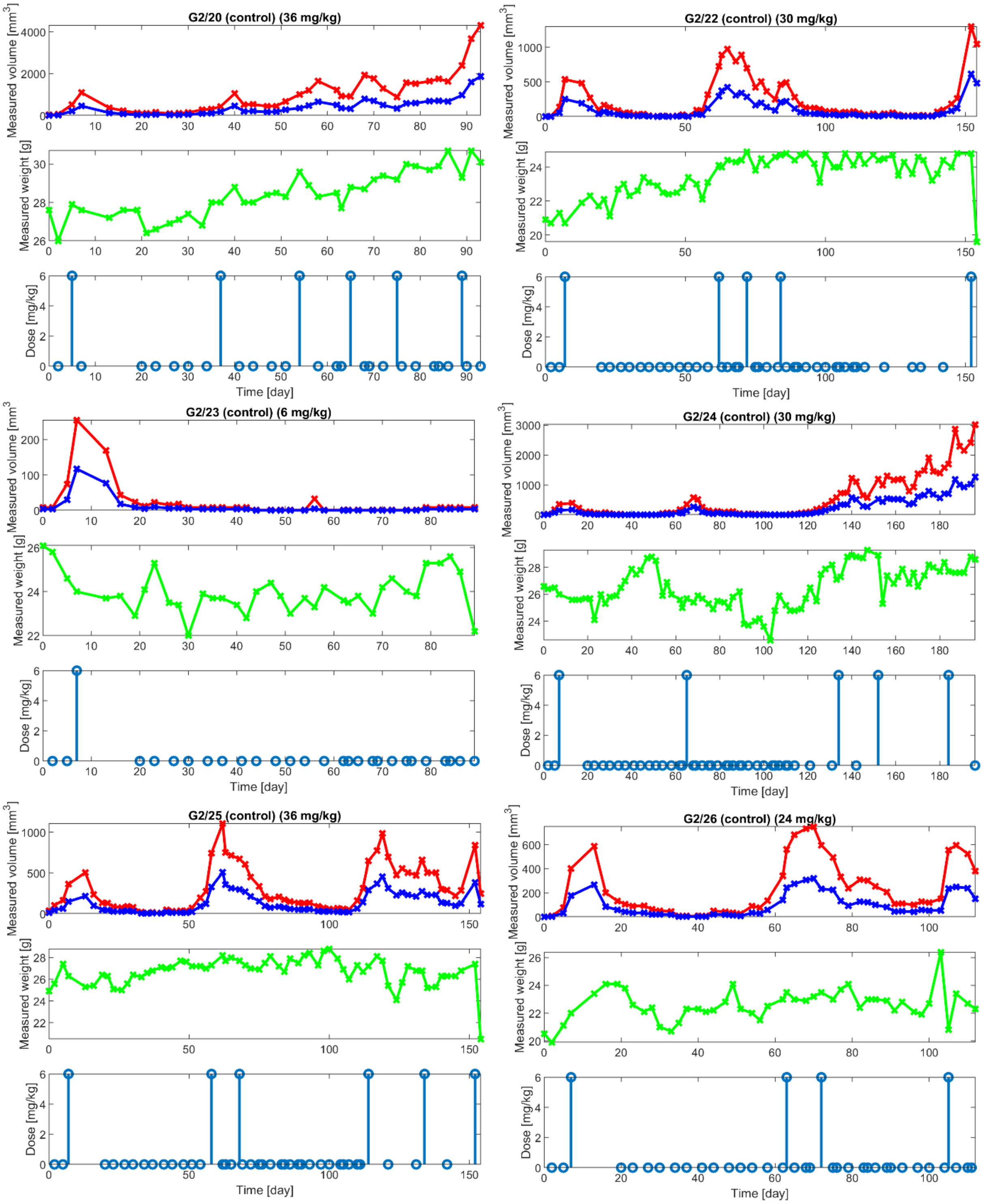

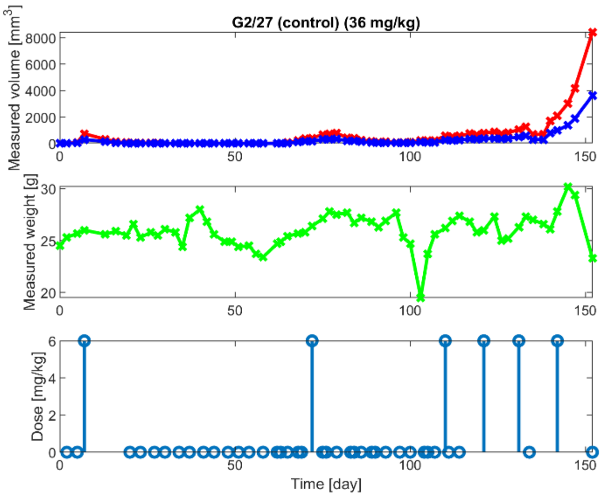
G2/27 (control) (36 mg/kg)
Measured volume [mm3]
Measured weight [g]
Dose [mg/kg]
Time [day]

b

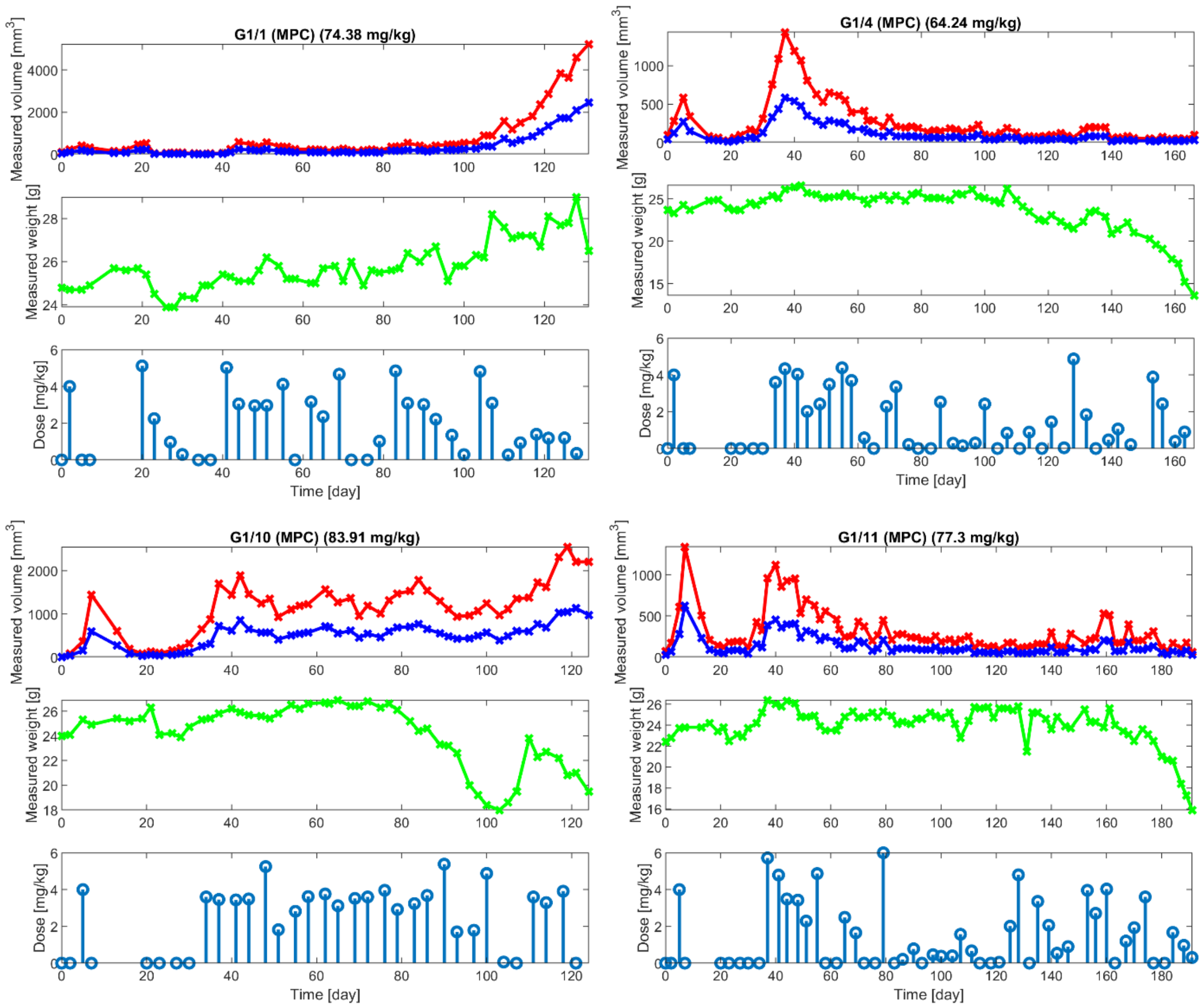
G1/1 (MPC) (74.38 mg/kg)
G1/4 (MPC) (64.24 mg/kg)
G1/10 (MPC) (83.91 mg/kg)
G1/11 (MPC) (77.3 mg/kg)
Measured volume [mm3]
Measured weight [g]
Dose [mg/kg]
Time [day]

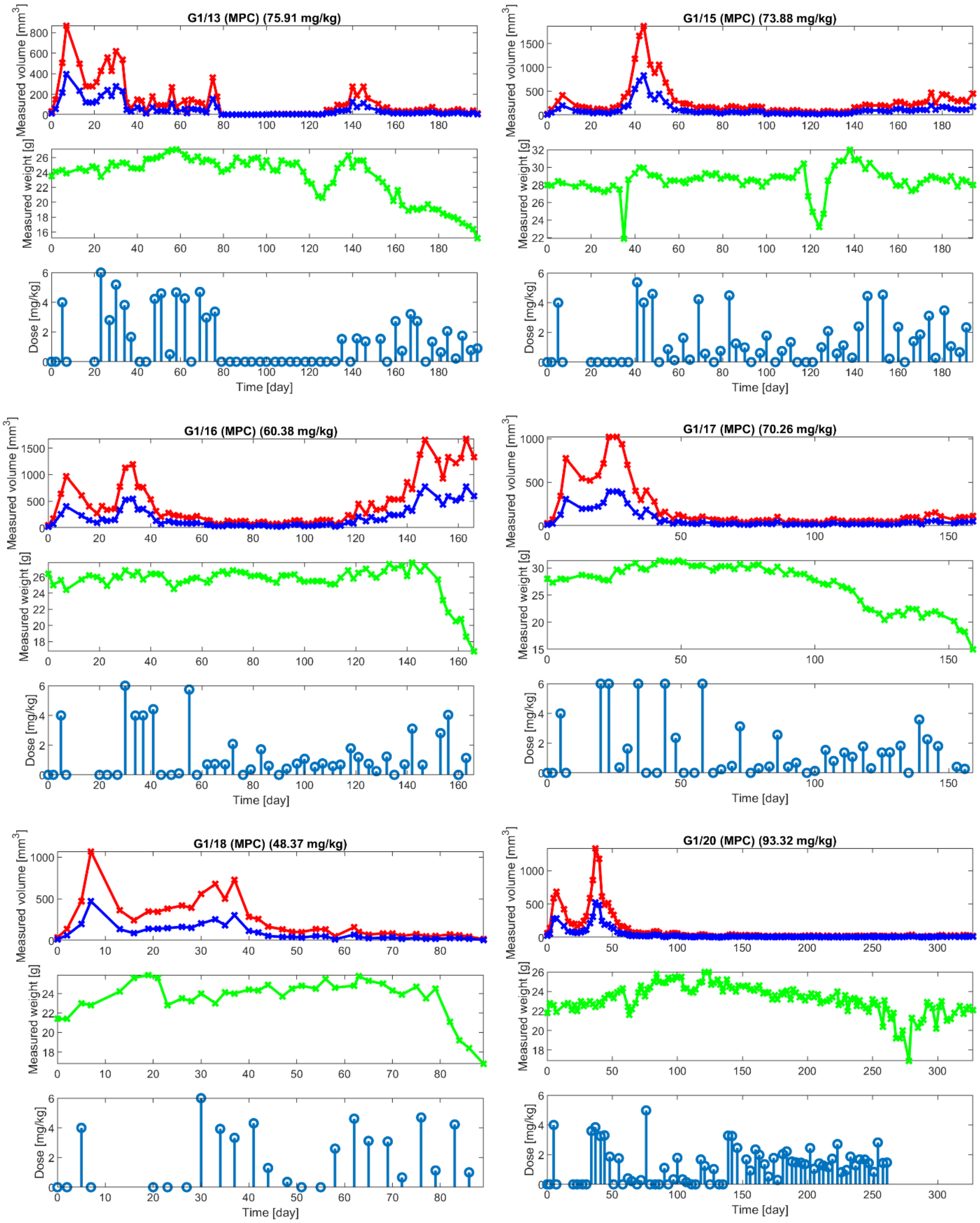
G1/13 (MPC) (75.91 mg/kg)
G1/15 (MPC) (73.88 mg/kg)
G1/16 (MPC) (60.38 mg/kg)
G1/17 (MPC) (70.26 mg/kg)
G1/18 (MPC) (48.37 mg/kg)
G1/20 (MPC) (93.32 mg/kg)
Measured volume [mm^3]
Measured weight [g]
Dose [mg/kg]
Time [day]

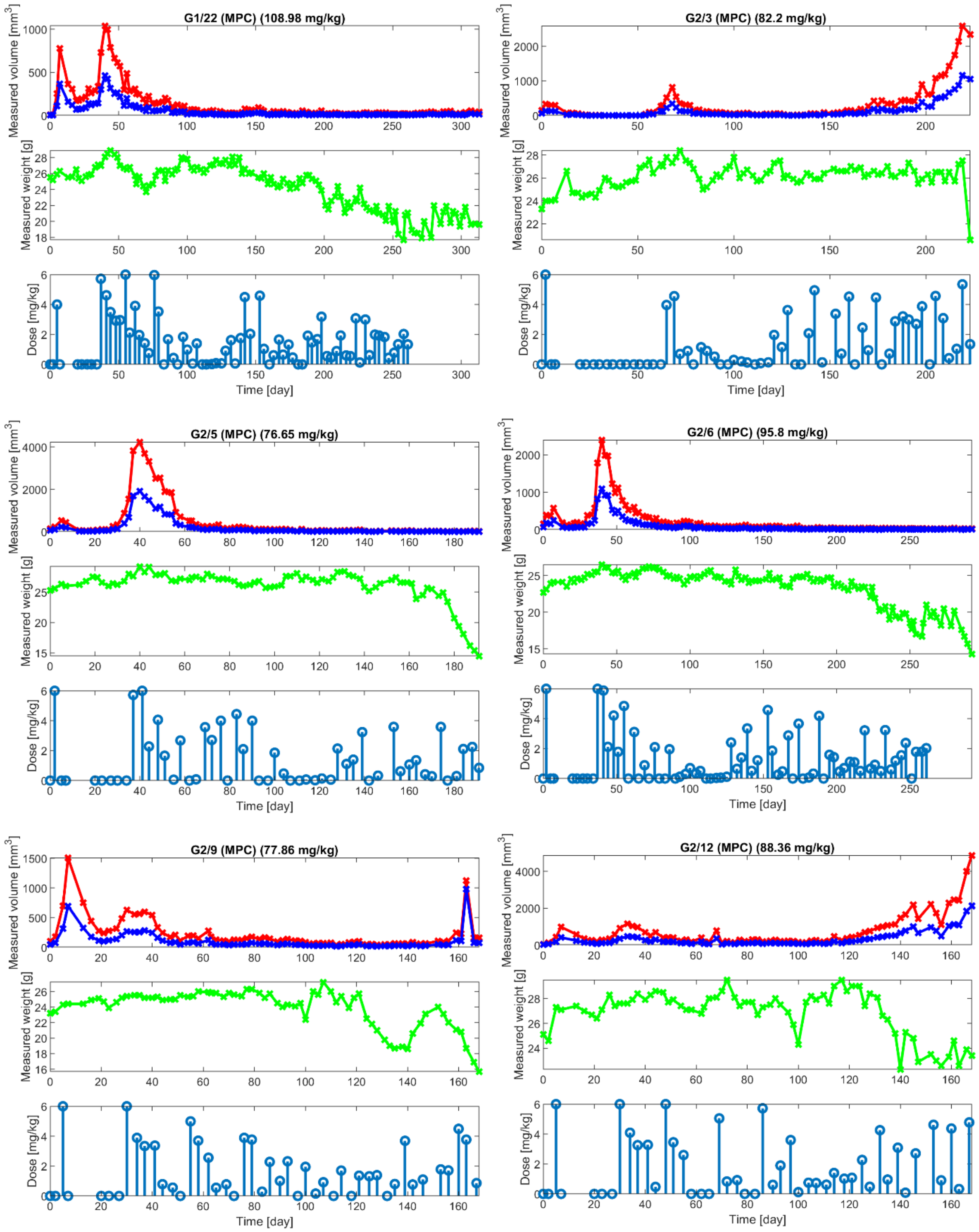
G1/22 (MPC) (108.98 mg/kg)
G2/3 (MPC) (82.2 mg/kg)
G2/5 (MPC) (76.65 mg/kg)
G2/6 (MPC) (95.8 mg/kg)
G2/9 (MPC) (77.86 mg/kg)
G2/12 (MPC) (88.36 mg/kg)
Measured volume [mm^3]
Measured weight [g]
Dose [mg/kg]
Time [day]

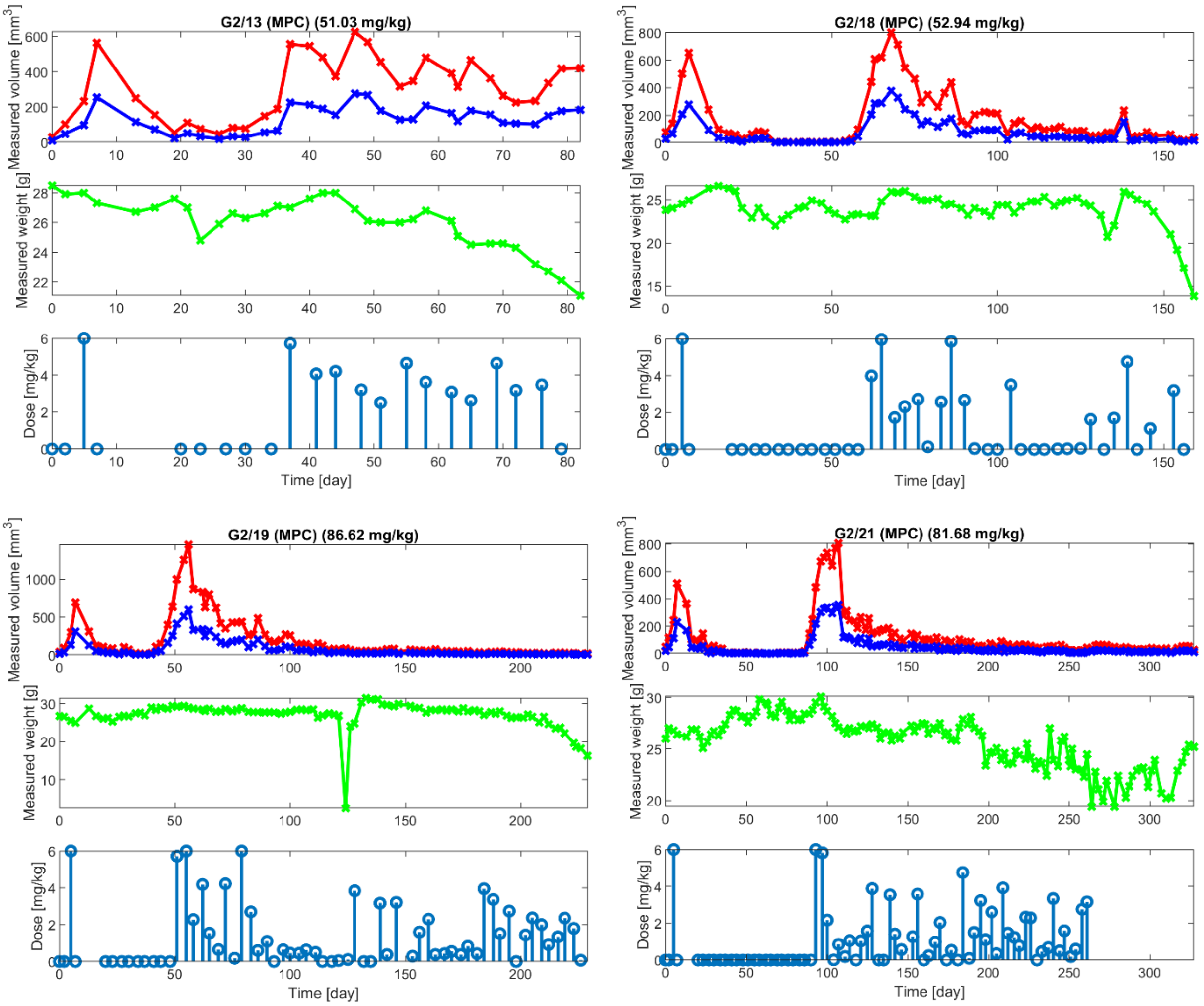
G2/13 (MPC) (51.03 mg/kg)
G2/18 (MPC) (52.94 mg/kg)
G2/19 (MPC) (86.62 mg/kg)
G2/21 (MPC) (81.68 mg/kg)
Measured volume [mm^3]
Measured weight [g]
Dose [mg/kg]
Time [day]

c

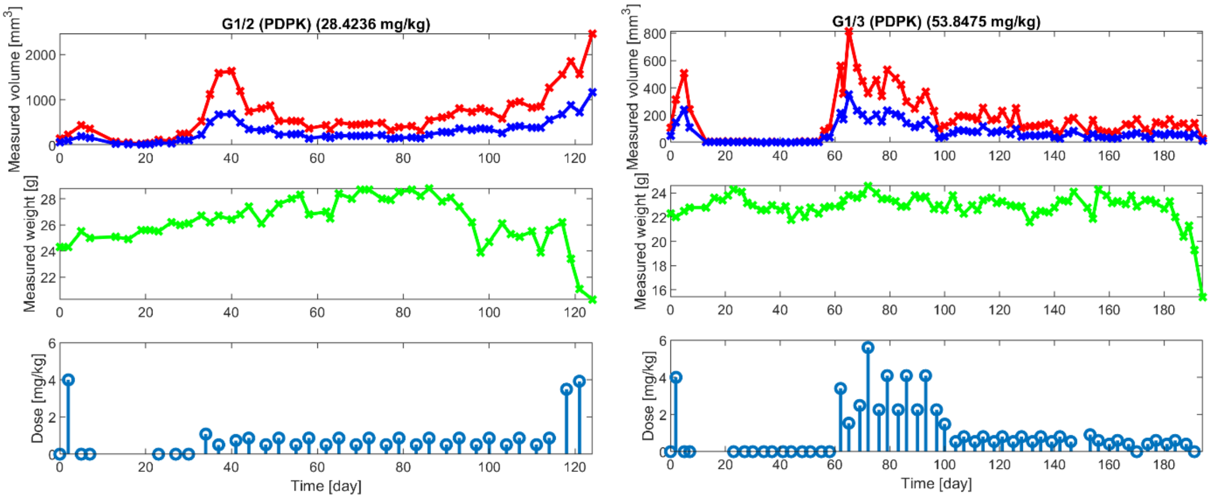
G1/2 (PDPK) (28.4236 mg/kg)
G1/3 (PDPK) (53.8475 mg/kg)
Measured volume [mm^3]
Measured weight [g]
Dose [mg/kg]
Time [day]

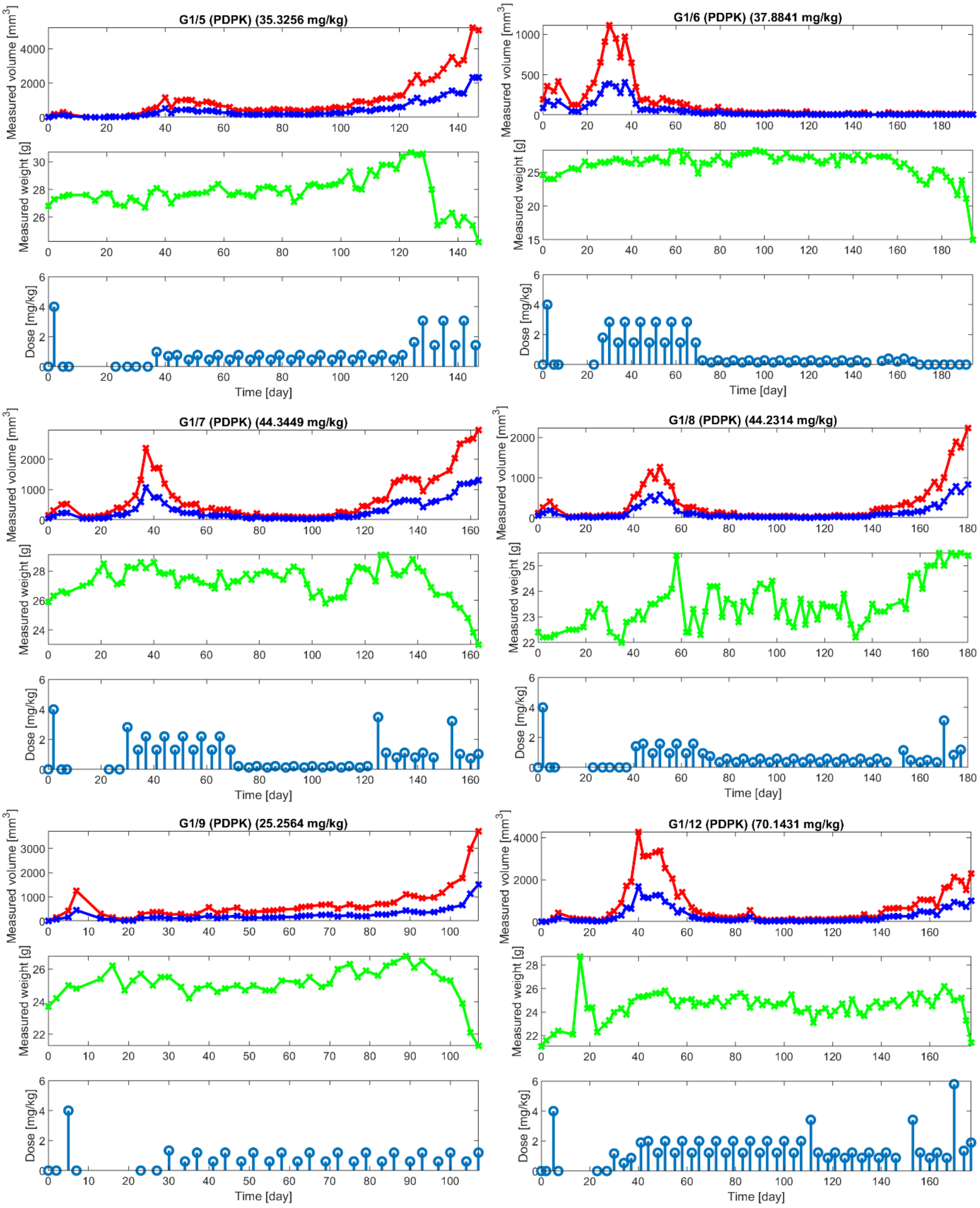
G1/5 (PDPK) (35.3256 mg/kg)
G1/6 (PDPK) (37.8841 mg/kg)
G1/7 (PDPK) (44.3449 mg/kg)
G1/8 (PDPK) (44.2314 mg/kg)
G1/9 (PDPK) (25.2564 mg/kg)
G1/12 (PDPK) (70.1431 mg/kg)
Measured volume [mm^3]
Measured weight [g]
Dose [mg/kg]
Time [day]

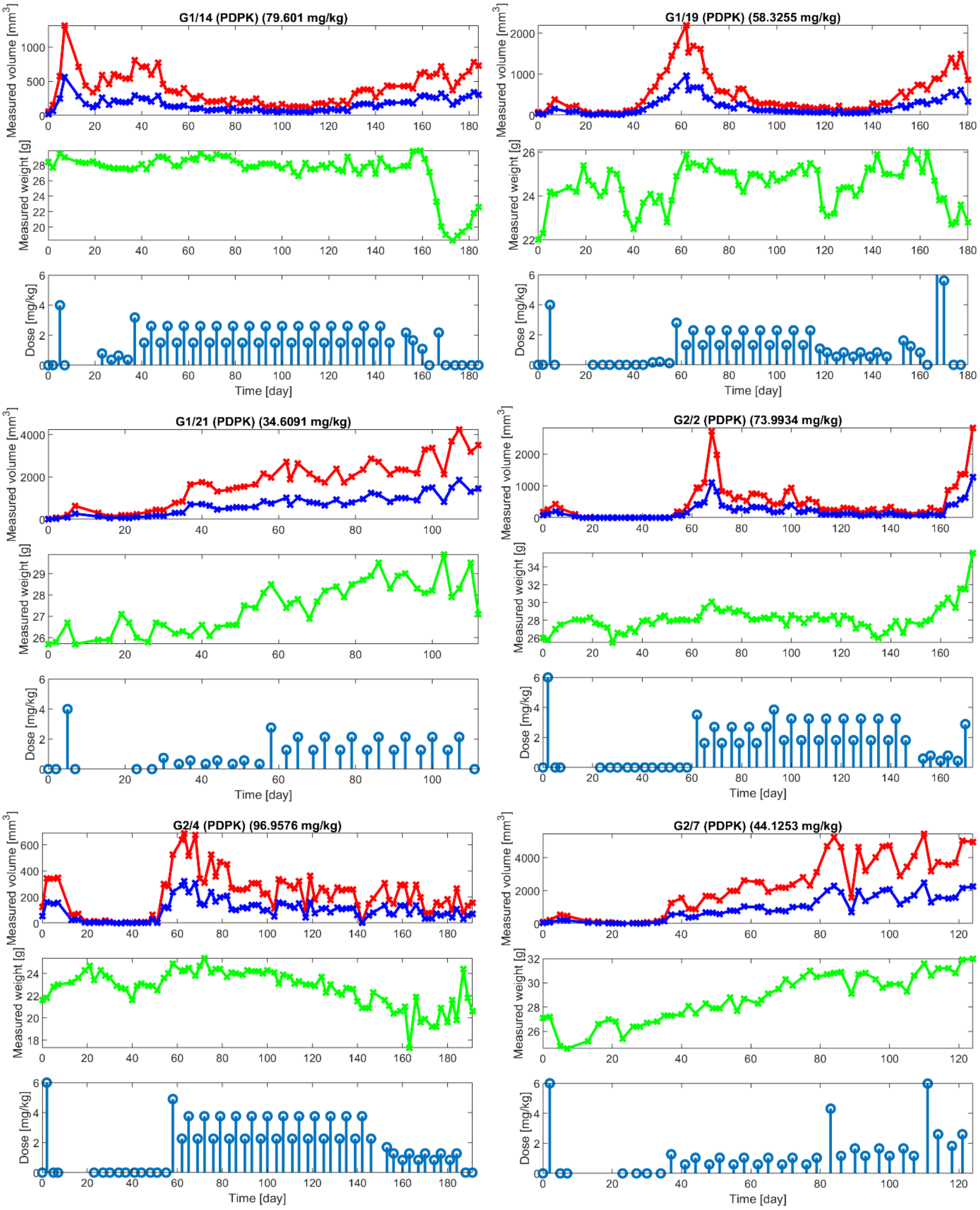
G1/14 (PDPK) (79.601 mg/kg)
G1/19 (PDPK) (58.3255 mg/kg)
G1/21 (PDPK) (34.6091 mg/kg)
G2/2 (PDPK) (73.9934 mg/kg)
G2/4 (PDPK) (96.9576 mg/kg)
G2/7 (PDPK) (44.1253 mg/kg)
Measured volume [mm^3]
Measured weight [g]
Dose [mg/kg]
Time [day]

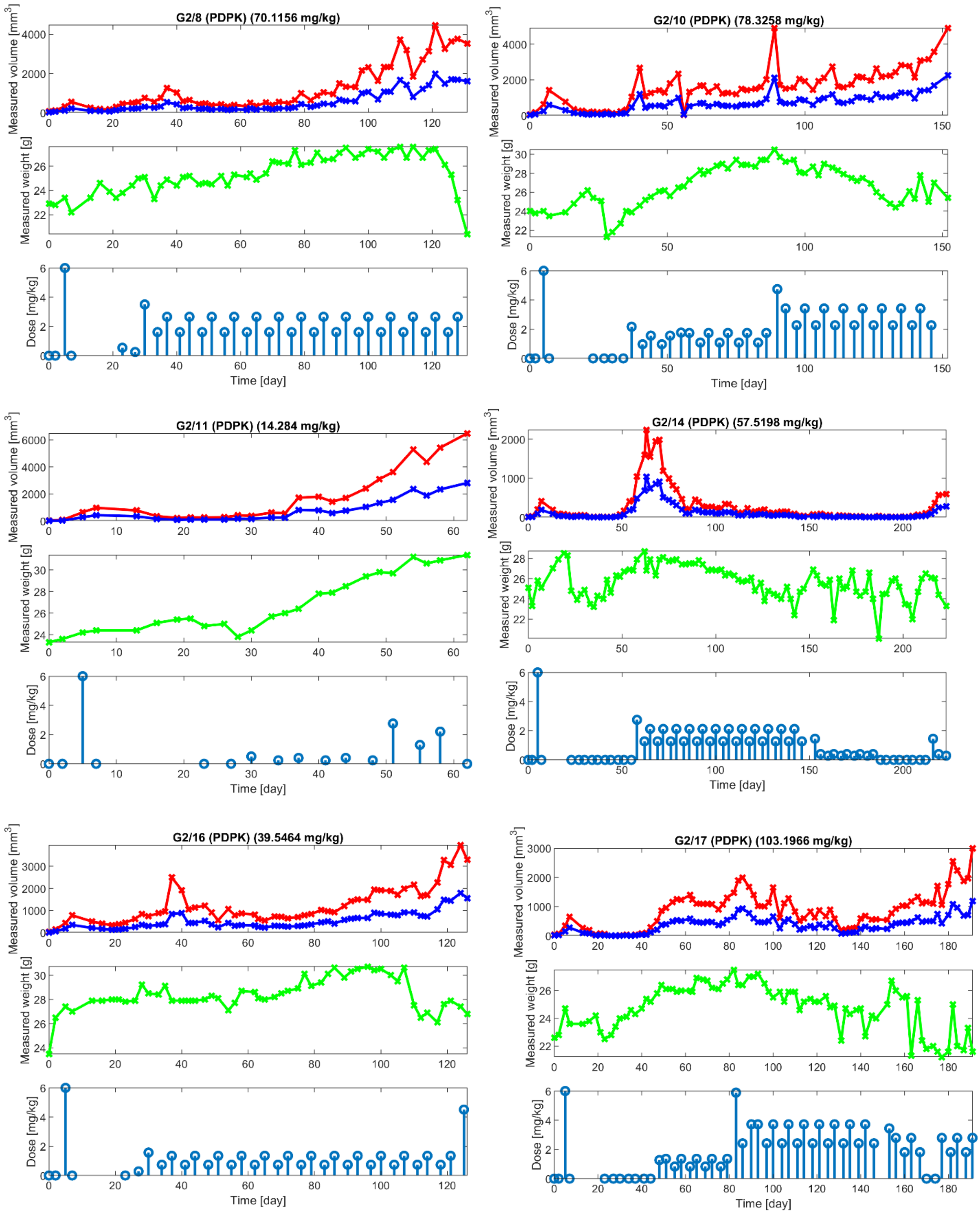

Supplementary Fig 4.: Redefined workflow results of AATD experiments **a** Representative growth kinetics data obtained from the tumor-triggered MTD treated group simulating conventional chemotherapy (control). The first plot shows the evolution of the tumor volume calculated by equation 1 (blue) or equation 2 (red). The second plot shows the evolution of the body mass of the mouse, and the last plot shows the standard and optimized doses.

b Growth kinetics data obtained from the group of MPC. The first plot shows the changes in tumor volume in the same manner as describe above. The second plot shows the evolution of mass of the mice, and the last plot shows the standard and optimized doses. The optimized therapy was based on the MPC.

c Growth kinetics data obtained from the group of PDPK. The 3 plots show the changes in tumor growth (upper), in body weight (middle) and the given PLD doses (lower). The optimized therapy was based on the PDPK.

References

1 Drexler, D. A., Ferenci, T., Füredi, A., Szakács, G. & Kovács, L. Experimental data-driven tumor modeling for chemotherapy. *IFAC-PapersOnLine* **53**, 16245–16250 (2020). https://doi.org/10.1016/j.ifacol.2020.12.619

2 Drexler, D. A., Ferenci, T., Lovrics, A. & Kovács, L. Tumor dynamics modeling based on formal reaction kinetics. *Acta Polytechnica Hungarica* **16**, 31–44 (2019).

3 Kovács, L. *et al.* Positive Impulsive Control of Tumor Therapy—A Cyber-Medical Approach. *IEEE Transactions on Systems, Man and Cybernetics: Systems* **54**, 597 - 608 (2024). https://doi.org/10.1109/TSMC.2023.3315637

4 Kovács, L. *et al.* Experimental Closed-Loop Control of Breast Cancer in Mice. *Complexity* **2022**, 10 (2022). https://doi.org/10.1155/2022/9348166
